# Supplementary material for: DIAMetAlyzer allows automated false-discovery rate-controlled analysis for data-independent acquisition in metabolomics
Source: Nat Commun. 2022 Mar 15;13:1347. doi: 10.1038/s41467-022-29006-z (PMC8924252; doi:10.1038/s41467-022-29006-z)
Supplement: Supplementary file 1 — Supplementary Information [file 41467_2022_29006_MOESM1_ESM.pdf]

Supplementary Information

DIAMetAlyzer allows automated false-discovery rate-controlled analysis for data-independent acquisition in metabolomics

Oliver Alka[1,2,*], Premy Shanthamoorthy[3,4], Michael Witting[5,6,7], Karin Kleigrewe[8], Oliver Kohlbacher[1,2,9], Hannes L Röst[3,4,10,*]

1 Applied Bioinformatics, Center for Bioinformatics, University of Tübingen, Tübingen, Germany
2. Institute for Bioinformatics and Medical Informatics, University of Tübingen, Tübingen, Germany
3 Terrence Donnelly Centre for Cellular & Biomolecular Research, University of Toronto, Toronto, Canada
4 Department of Molecular Genetics, University of Toronto, Toronto, Canada
5 Metabolomics and Proteomics Core, Helmholtz Zentrum München, Neuherberg, Germany
6 Research Unit Analytical BioGeoChemistry, Helmholtz Zentrum München, Neuherberg, Germany
7 Chair of Analytical Food Chemistry, School of Life Sciences Weihenstephan, Technical University of Munich, Freising, Germany
8 Bavarian Center for Biomolecular Mass Spectrometry (BayBioMS), Technical University of Munich, Freising, Germany
9 Institute for Translational Bioinformatics, University Hospital Tübingen, Tübingen, Germany
10 Department of Computer Science, University of Toronto, Toronto, Canada
email: oliver.alka@uni-tuebingen.de; hannes.rost@utoronto.ca

Table of Contents

Supplementary Information *1*

Targeted extraction, scoring and FDR estimation 3

PyProphet model performance 4

Combining identification information between collision energies 5

Optimizing for unique identifications 6

Variable SWATH windows 7

Dilution series 8

FDR calibration at different collision energies 9

Quantification behavior of detected metabolites 9

Limit of detection 11

Quantification behavior at higher collision energy ranges 17

Comparison of identification performance between DIAMetAlyzer and MS-DIAL 18

Additional methods for the comparison with MetaboDIA using the AMD dataset 19

Combining assay libraries 20

Library comparison (MetaboDIA vs DIAMetAlyzer) 21

The difference in DDA feature detection and linking of MetaboDIA and DIAMetAlyzer 22

Targeted vs non-targeted quantification (MetaboDIA vs DIAMetAlyzer) 26

Patient group comparison of the AMD data 27

Differential expression analysis of AMD data 28

Quantification and evaluation of AMD biomarkers and additional candidates 28

Further decoy generation methods and evaluation 31

Comparison of DDA and DIA data 33

Example workflow 34

Evaluation of the identification performance 36

Supplementary References 38

Supplementary Note *41*

Software Requirements 41

Targeted extraction, scoring and FDR estimation

The false discovery rate (FDR) was introduced, handling the results of the increasing amounts of genomics data. It is the expected ratio of false-positive classifications (false discoveries) to the total number of positive classifications.

FDR = FP / (FP+TP)

In this context, "discovery" is a statistical term rooted in the context of positives and negatives. In our context, the "positives" would be true quantification events (e.g. a signal in the XIC that corresponds to the analyte of interest) while the "negatives" would be false quantification events (e.g. a signal / region in the XIC that does not correspond to the analyte of interest and is either chemical noise or belongs to another analyte). In original terminology as introduced in 1995, the "discovery" stands for the items that are labeled as "positive", and hence could be true positives or false positives, in a gene expression experiment as the genes that you label as differentially expressed[1].

In 2007, Elias et al. introduced the concept of target-decoy FDR in mass spectrometry-based proteomics[2], where it is used to distinguish correct from incorrect peptide identifications.

Unfortunately, creating plausible decoys in metabolomics is not as straightforward since methods like shuffling or reversing a sequence do not work in metabolomics. However, target-decoy based FDR estimation was introduced recently for large scale untargeted metabolomics studies[3,4]. Scheubert et al. presented methods to ensure the consistency of the decoy spectra by using fragmentation tree re-rooting[3]. First, a fragmentation tree is constructed for the original spectrum identification assigning fragments compatible with the metabolite substructures. In a second step, this fragmentation tree will be re-rooted. A new root is chosen, leading to tree rearrangements by shifting the fragmentation reaction order. The results are new potential fragments based on the original metabolite. The generated decoys should have a similar probability of occurring in the sample and could represent the same metabolite - a decoy. The FDR for hits in a spectral library database can be estimated with this method.

In the targeted setting, the peak group FDR estimation works differently. Experimental specific targets and decoys are added to the assay library (prior knowledge database) used for targeted extraction. For available targets and decoys, peak groups are extracted and scored (Supplementary Fig. 1). A discriminant score (d-score) distribution is computed using a linear discriminant analysis based on the available subscores, and statistical error estimates are derived by fitting a null distribution[5–7] (Supplementary Fig. 2).

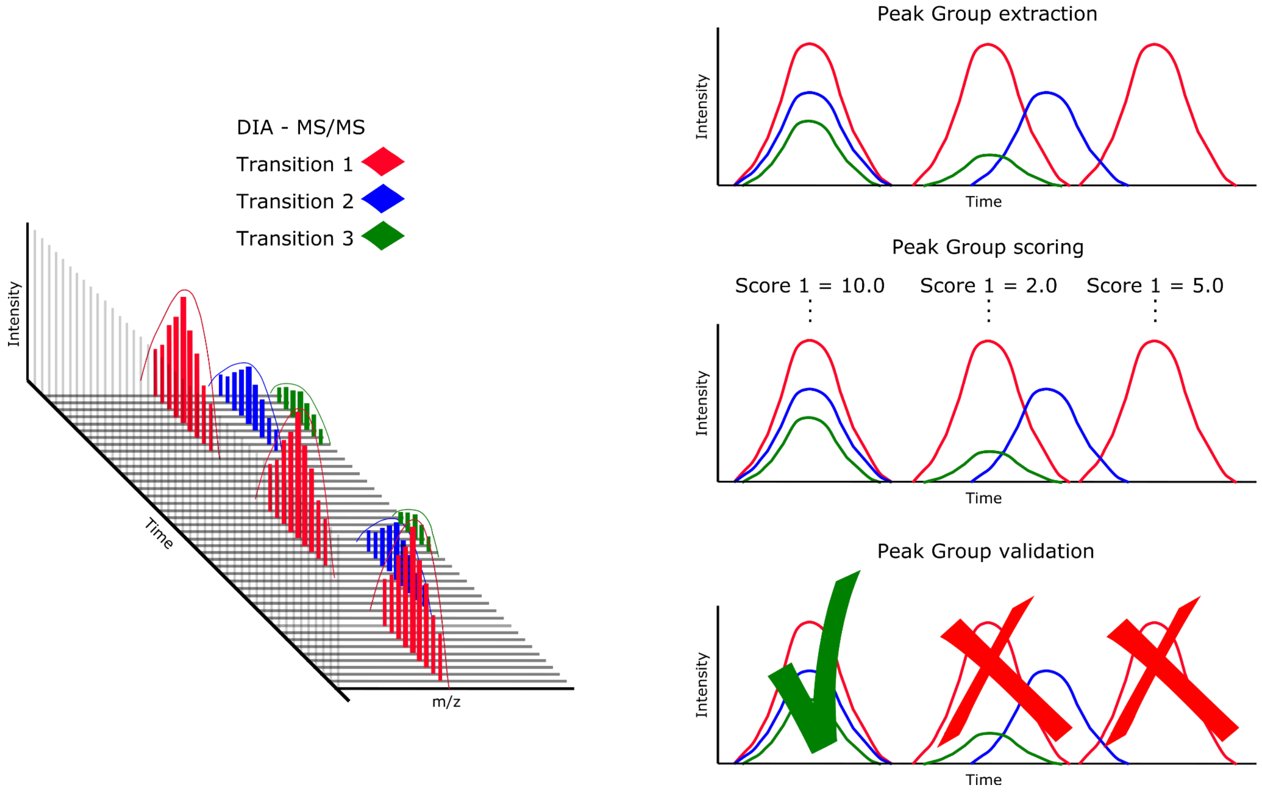

Supplementary Figure 1 | Targeted extraction and scoring. Three transitions belonging to an analyte are extracted from DIA MS/MS data in a defined retention time window. Peak group candidates are extracted as chromatograms, scored and validated.

In terms of FDR control, the importance or span of the FDR depends on the research question. For example, a low number of false positives in clinical biomarker identification is important (1% - 5% FDR). On the other hand, if the aim is to find new potential biomarkers, an FDR of 10% might still be valid since these must be further validated.

PyProphet model performance

Performance of the PyProphet[6,7] linear discriminant analysis for metabolomics data. For MS1 and MS2 scoring scores, with a low cross-correlation, were used. Using semi-supervised learning, a combined discriminant score was established. The group discriminant-score (d-score) distribution and density show the decoy and target population results (Supplementary Fig. 2a,b). Decoy and false targets are nicely separated from the true targets, based on the d-score scoring. The p-value density histogram shows an anti-conservative p-value distribution estimated based on the Storey method[8].

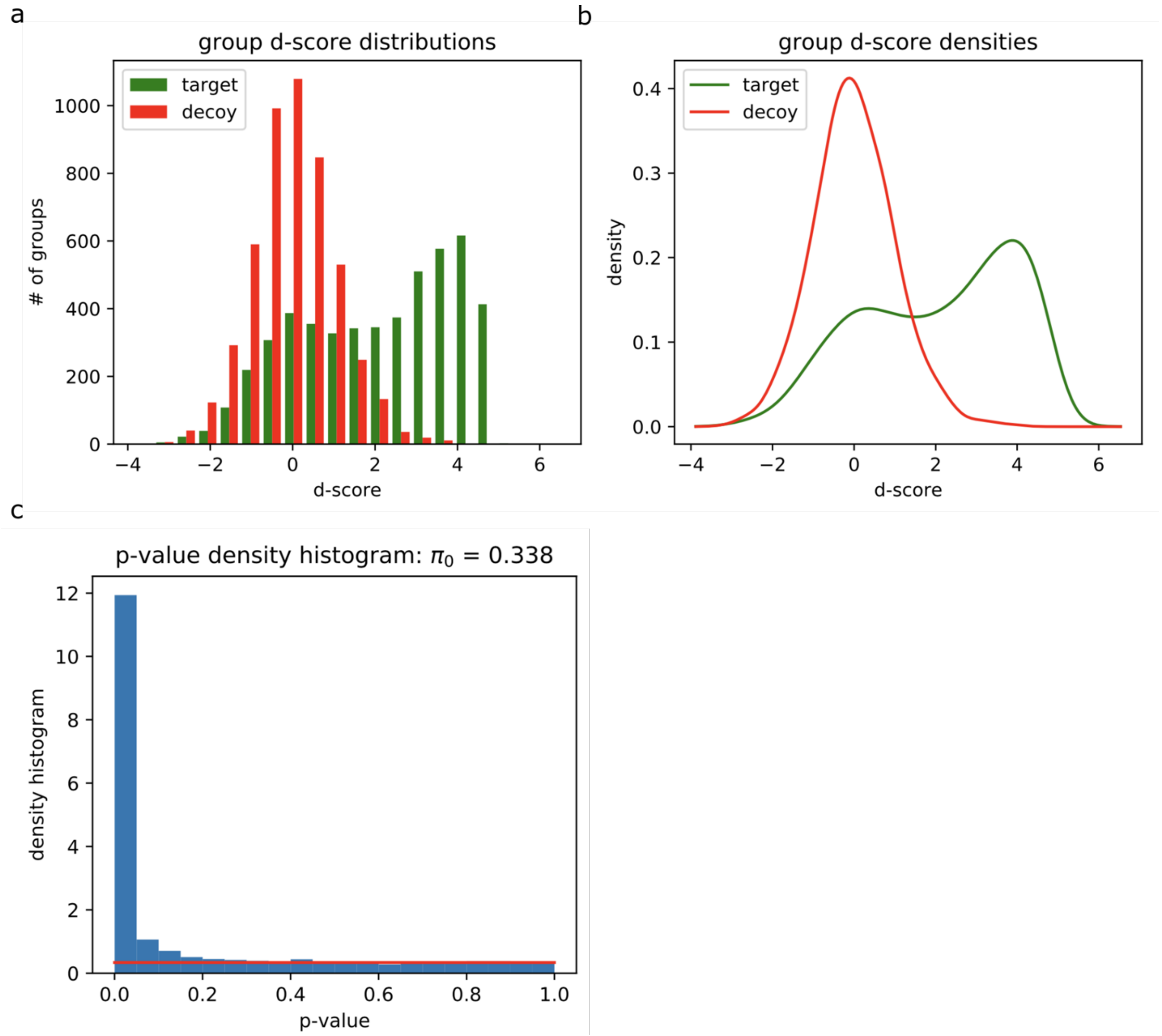

Supplementary Figure 2 | PyProphet target decoy evaluation. a,b) The group discriminant-score (d-score) distribution and density show the decoy and target population results. Decoy and false targets are nicely separated from the real targets, based on the d-score scoring. c) The p-value density histogram showing anti-conservative p-value distribution estimated based on the Storey method.

Combining identification information between collision energies

Depending on the molecular mass and structure of the metabolites the fragmentation behavior may differ, depending on the collision energy. Multiple collision energies or collision energy ranges may boost library performance[9]. Here, combining the two collision energy ranges (20-50 eV & 50-80 eV) can boost assay library coverage by 11% (Supplementary Fig. 3).

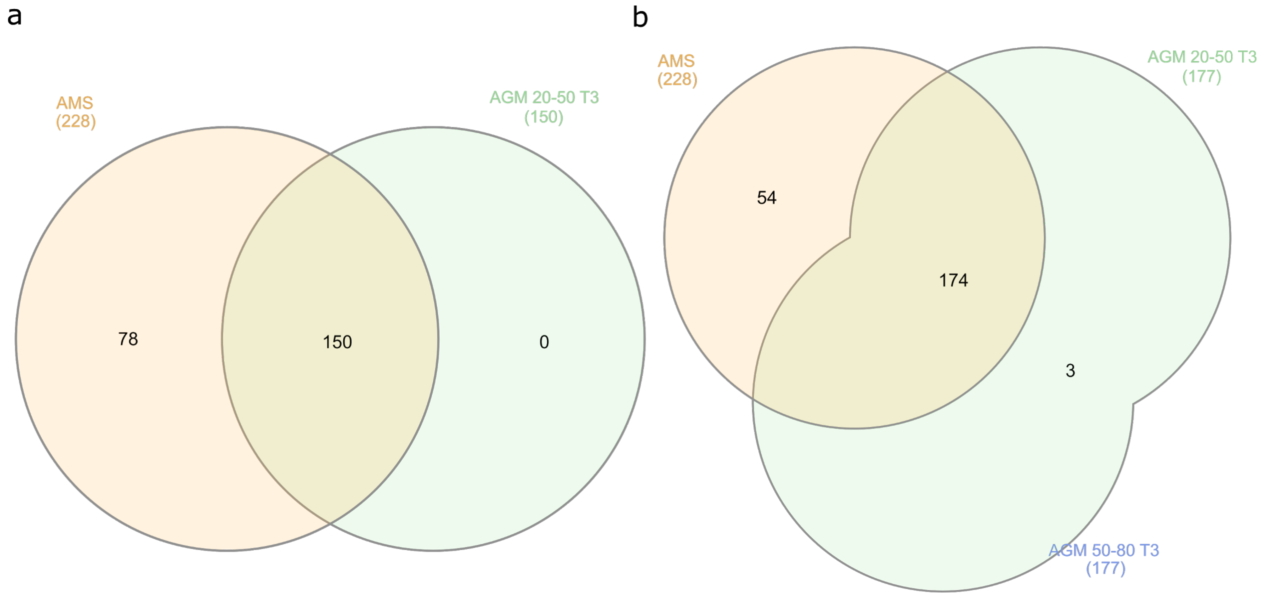

Supplementary Figure 3 | Library coverage. Identification at MS1 level was performed using AccurateMassSearch (AMS). The library with fragment annotation at MS2 level and 3 transitions (T3) was generated using the AssayGeneratorMetabo (AGM). a) Library generation using 20-50 eV data has a coverage of 66% detected and identified compounds. b) Combining detected and identified compounds from both collision energy ranges (20-50 eV, 50-80 eV) increases the coverage of the target-decoy library to 77%.

Optimizing for unique identifications

To investigate the effects of assay redundancy and specificity within our assay library, we first used computational models to calculate nonredundant theoretical assays, also known as unique ion signatures (UIS), for a given metabolomic background[10–12]. UIS_n is defined as a set of top *n* mass-to-charge values (precursor and fragment *m/z*) that maps exclusively to one metabolite in the metabolome to be analyzed[22]. For this analysis, we simulated classic MS methods (MS1, MRM, SWATH) using the assay library and NIST 17 LC/MS as a combined background with varying mass accuracy for both the precursor *m/z* window (MS1) and the fragment *m/z* window (MS2) to compare differences between UIS_1, UIS_2 and UIS_3. This analysis was performed for data acquired in both low (20-50 eV) and high (50-80 eV) ranges of collision energy. This analysis demonstrates that SWATH outperforms MS1-only analyses, while performing comparably to MRM-based analyses when using high accuracy fragment ion information only (33.2%, 56.1% and 62.8% unambiguous compounds respectively for MS1-only, SWATH Variable Windows/25 ppm and MRM UIS_3, Supplementary Fig. 4a). However, with increased MS1 accuracy, SWATH outperforms MRM-based analyses (with a few transitions) due to its capability of extracting both high-resolution MS1 precursor ion traces as well as high-resolution fragment ion traces for any analyte of interest (93.4% unambiguous compounds for SWATH 25pm/25ppm UIS_3, simulating both MS1 and MS2 XIC analysis, Supplementary Fig. 4a, rightmost bars). Based on these overall results and our previous study[13], scoring is based on both MS1 and MS2 information, in addition to retention time and fragment ion relative intensity .

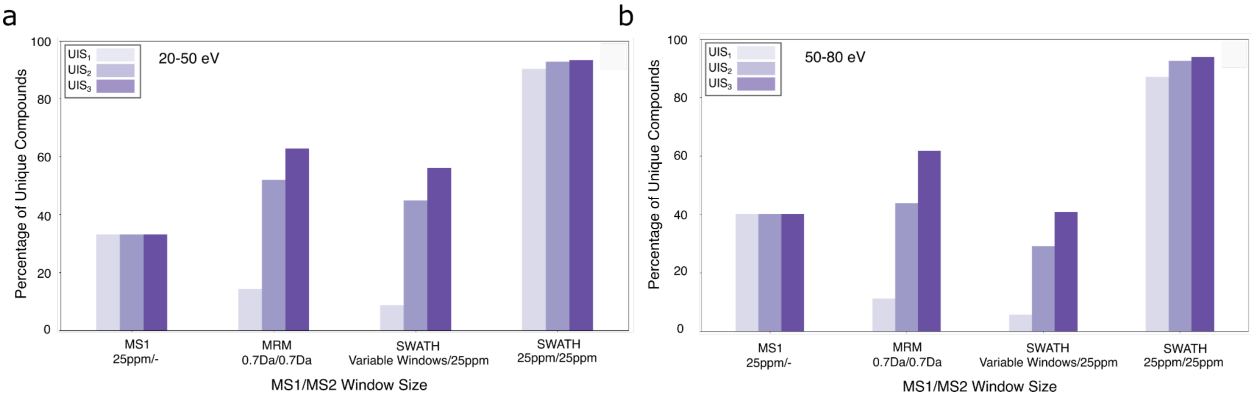

Supplementary Figure 4 | Percentage of Unique Compounds for different MS methods by comparison of UIS1, UIS2 and UIS3. Using the assay library and the NIST 17 LC/MS database as background, simulations were conducted for each analyte (query) by setting different mass tolerances associated with different MS methods. The following MS1/MS2 windows were set: MS1-only: 25ppm/-; Multiple Reaction Monitoring (MRM): 0.7 Da/0.7 Da; and Data-Independent Acquisition (DIA/SWATH): Variable Windows/25 ppm, 25ppm/25ppm. The percentage of compounds in the assay library with no interference (the background for each query based on the given parameters), known as the percentage of unique compounds (y-axis), is calculated for each method (x-axis) a) for data acquired at collision energy ranges of 20-50 eV b) and 50-80 eV.

Variable SWATH windows

The variable SWATH windows were assessed based on the plasma matrix using the SWATH Variable Window Calculator (SCIEX). The target number of windows was set to 8, lower and upper m/z limit to 100 and 900, respectively. "Round bin edges to x figures" was set to 1 and a window overlap of 1 Da was chosen. In addition, the minimum window was set to 3 and CES to 5. With these settings, the windows in Supplementary Table 1 were determined.

Supplementary Table 1 | Variable SWATH windows assessed using SWATH Variable Window Calculator on the plasma matrix

Start m/z	End m/z
99.5	157.7
156.7	242.6
241.6	370.3
369.3	465.9
464.9	511.7
510.7	557.5
556.5	627.7
626.7	886.1

Dilution series

The concentration of the 250 pesticides over the 1:4 dilution series (Supplementary Table 2). The concentration of the metabolite is dependent on its molecular mass, which ranges from 141 to 874 g/mol. Using an injection volume of 5 µL the concentration would range from the highest concentration (step1 - min: 5717.501 fmol/µL, max 35460.666 fmol/µL) to the lowest concentration (step 10 - min: 0.022fmol/µL, max 0.135 fmol/µL) covering 5 orders of magnitude (Supplementary Fig. 5).

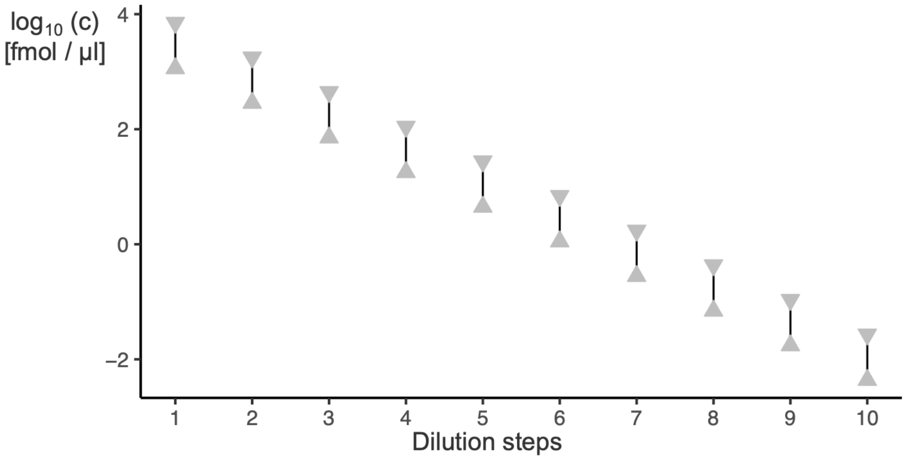

Supplementary Figure 5 | Concentration of the pesticides over the dilution series. The concentration gradient over the dilutions series covers 5 orders of magnitude and depends on the molecular mass of the pesticide. Covering minimum 5717.501 fmol/µL (step 1) to 0.022fmol/µL and maximum 35460.666 fmol/µL to 0.135 fmol/µL (step 10) over the 1:4 dilution series.

Supplementary Table 2 | Preparation of the 1:4 dilution series of the pesticide mix in blood plasma measured via SWATH acquisitions.

Step	Dilution	Previous dilution (µL)	Plasma matrix (µL)	Replicates
1	1	-	-	3
2	4	25	75	3
3	16	25	75	3
4	64	25	75	3
5	256	25	75	3
6	1024	25	75	3
7	4096	25	75	3
8	16384	25	75	3
9	65536	25	75	3
10	262144	25	75	3

FDR calibration at different collision energies

The FDR based on the fragmentation tree re-rooting approach has a conservative tendency, with a slight overestimation for data acquired at lower ranges of collision energy (20-50 eV). In comparison, FDR estimates for data acquired at higher ranges of collision energy (50-80 eV) demonstrated an increased abundance of overlapping fragments resulting in more complicated analyses (Supplementary Fig. 6). We determined the precision and recall based on different estimated FDR thresholds using the best peak group rank, to verify the accuracy of the classifier. The area-under-the-curve is 96% and 93%, respectively.

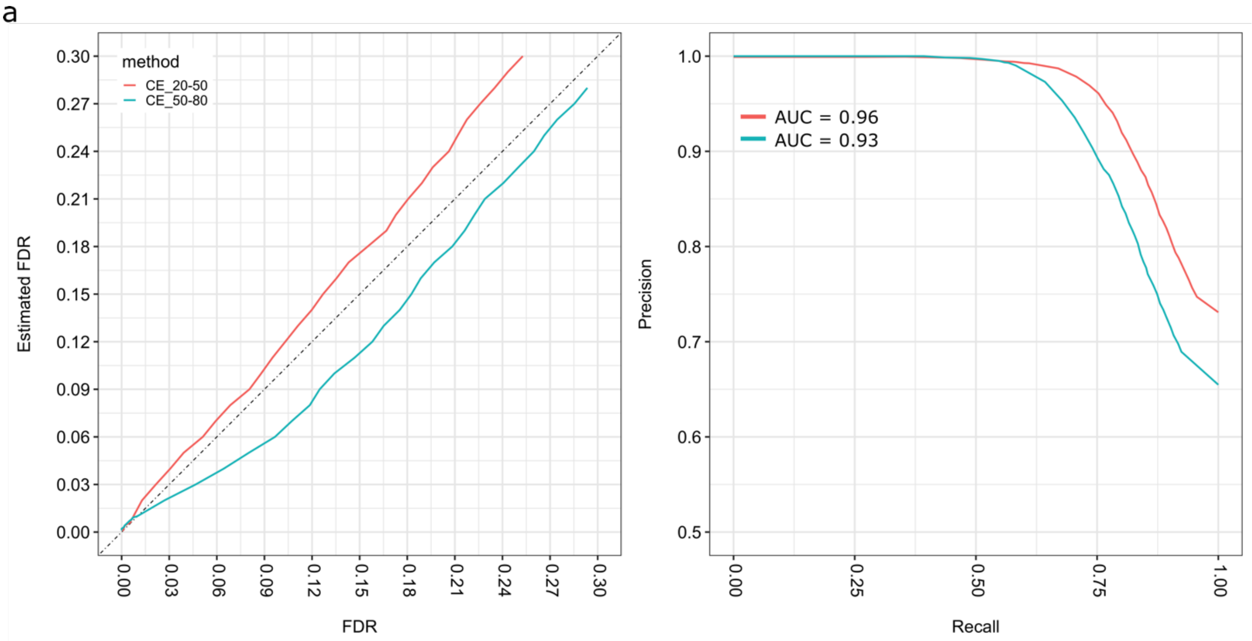

Supplementary Figure 6 | Identification accuracy of the DIAMetAlyzer on the pesticide spike-in dataset at different collision energies. a) The estimated FDR versus the ground truth FDR (20-50 eV, red; 50-80 eV, blue). The continuous line at 45 degrees shows the optimal values. b) Precision-Recall curve with the area-under-the-curve (AUC (20-50 eV, red) = 0.96 , AUC (50-80 eV, blue) = 0.93).

Quantification behavior of detected metabolites

Three different quantification behaviors were determined, illustrated by the following metabolites (Supplementary Fig. 7). Amidosulfuron (exact mass: 369.04 Da) has a low initial intensity and was detected in the first two dilutions. Azaconazole (exact mass: 229.02 Da), which had a higher measurable initial intensity was detected to a dilution of 1:1024. Fenpyroximate (exact mass: 421.20 Da), with the highest initial intensity, was detected to a dilution of 1:262144. Depending on the initial measurable intensity, the pesticides could be detected in samples with a higher dilution.

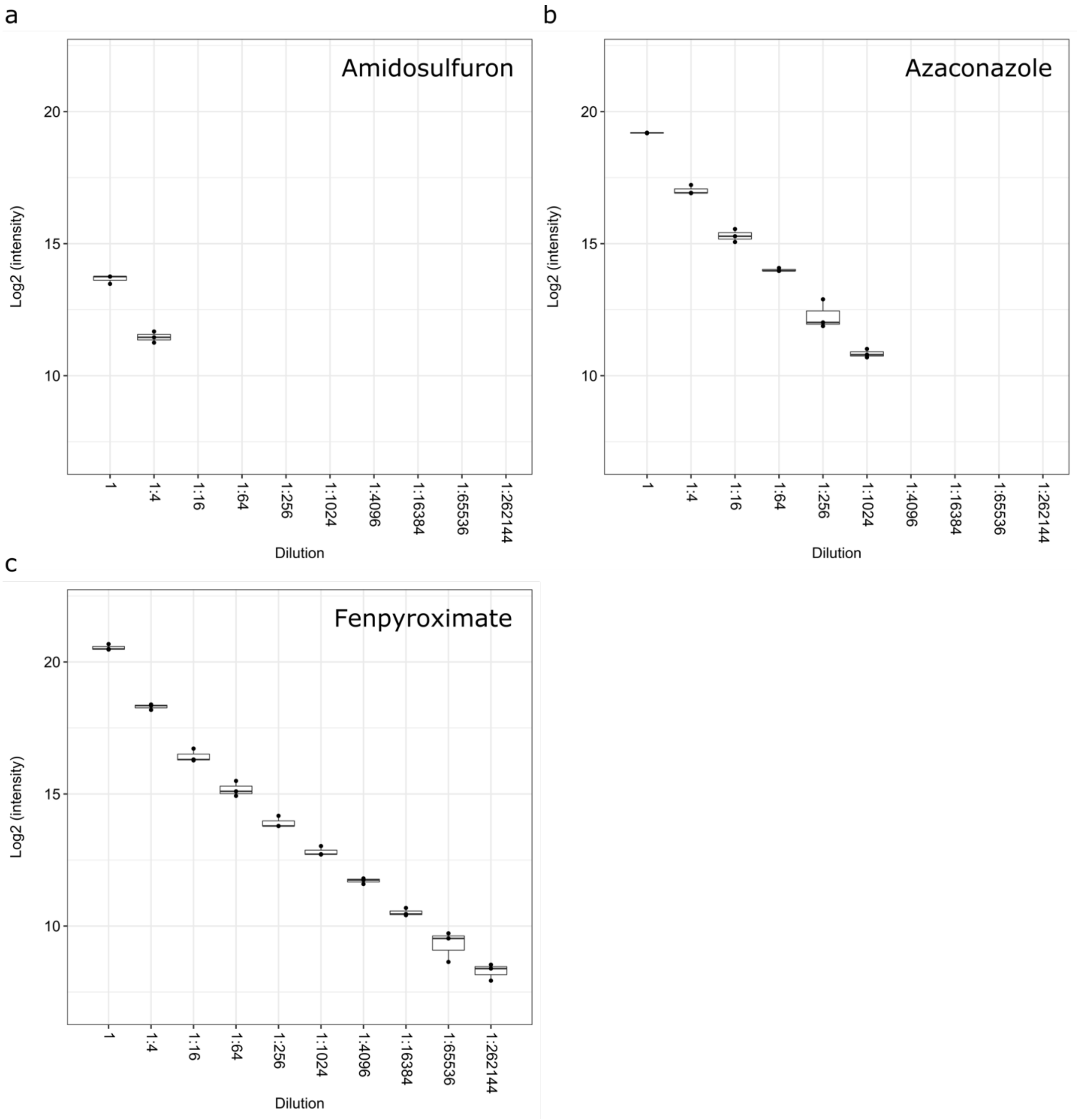

Supplementary Figure 7 | Quantification behaviors over the dilution series for different pesticides. a) Amidosulfuron (exact mass: 369.04 Da) has a low initial intensity and was detected in the first two dilutions. b) Azaconazole (exact mass: 229.02 Da) had a higher initial measurable intensity and was detected to a dilution of 1:1024. c) Fenpyroximate (exact mass: 421.20 Da) was detected to a dilution of 1:262144 and had the highest initial intensity. a-c) indicate median, 25th and 75th percentiles (middle line, Q1 and Q3 within the box respectively), including 1.5x interquartile range whiskers. Visualization of outliers were disabled to demonstrate the overlay with a dot plot to depict the underlying distribution (n per dilution = 3 technical replicates – benchmark dataset).

Limit of detection

We calculated the LOD, using the unfiltered results and the definition of (LOD = S/N > 10) for each pesticide (Supplementary Table 3). We used the intensity at the lowest dilution the compound was still detected via targeted extraction as noise. From there we calculated the concentration based on the exact mass, the initial concentration of 1 ng/µl and the corresponding dilution of the compound. Please see lod.RMD at https://github.com/oliveralka/DIAMetAlyzer_additional_code for details.

Supplementary Table 3 | Limit of detection

compound name	molecular formular	last SN over threshold	last dilution over threshold	LOD [fmol/μL]
Acephate	C4H10NO3PS	15	3	342
Sulfadiazole	C7H12N4O3S2	15	7	237
Carboxin	C12H13NO2S	11	7	266
Flusilazole	C16H15F2N3Si	20	5	198
Phosmet	C11H12NO4PS2	44	4	197
Thifensulfurson methyl	C12H13N5O6S2	23	6	161
Desmedipham	C16H16N2O4	14	7	208
Malaoxon	C10H19O7PS	17	6	199
Metribuzin	C8H14N4OS	14	6	292
Nitenpyram	C11H15ClN4O2	14	4	231
Dimethomorph	C21H22ClNO4	14	6	161
Fenamiphos	C13H22NO3PS	22	2	206
Metsulfuron-methyl	C14H15N5O6S	13	6	164
Trietazin	C9H16ClN5	19	7	273
Halofenozide	C18H19ClN2O2	11	5	189
Myclobutanil	C15H17ClN4	29	5	217
Halosulfuron-methyl	C13H15ClN6O7S	15	4	144
Hexaconazole	C14H17Cl2N3O	20	7	200
Iprovalicarb	C18H28N2O3	14	6	195

Triasulfuron	C14H16ClN5O5S	13	7	156
Azoxystrobin	C22H17N3O5	24	6	155
Bifenazate	C17H20N2O3	19	3	208
Fluopicolide	C14H8Cl3F3N2O	21	8	164
Metconazole	C17H22ClN3O	24	6	196
Rimsulfuron	C14H17N5O7S2	13	6	145
Zoxamide	C14H16Cl3NO2	15	6	187
Bifenazate	C17H20N2O3	79	9	208
Clethodim	C17H26ClNO3S	14	2	174
Isoprothiolane	C12H18O4S2	27	8	215
Methoprotryne	C11H21N5OS	15	7	231
Fenamidone	C17H17N3OS	12	5	201
Fluoxastrobin	C21H16ClFN4O5	12	8	136
Isoprothiolane	C12H18O4S2	23	7	215
Isoxaflutole	C15H12F3NO4S	12	3	174
Propioconazole	C15H17Cl2N3O2	11	7	183
Secbumeton	C10H19N5O	11	7	278
Amidosulfuron	C9H15N5O7S2	12	7	169
Bispyribac sodium salt	C19H18N4O8	12	6	138
Flufenacet	C14H13F4N3O2S	11	8	172
Fluoxastrobin	C21H16ClFN4O5	30	1	136
Triadimefon	C14H16ClN3O2	17	5	213
Azinphos-Ethyl	C12H16N3O3PS2	10	4	181
Chlorfenvinphos	C12H14Cl3O4P	21	7	175
Cyprodinil	C14H15N3	11	6	278
Furathiocarb	C18H26N2O5S	21	7	164

Methiocarb	C11H15NO2S	24	3	278
Methoxyfenozide	C22H28N2O3	19	3	170
Tribenuron methyl	C15H17N5O6S	25	4	158
Aminocarb	C11H16N2O2	80	9	300
Lenacil	C13H18N2O2	28	1	267
Tricyclazol	C9H7N3S	12	6	331
Dimoxystrobin	C19H22N2O3	14	7	192
Flazasulfuron	C13H12F3N5O5S	14	6	154
Ipconazole	C18H24ClN3O	17	5	188
Methoxyfenozide	C22H28N2O3	16	3	170
Propyzamide	C12H11Cl2NO	13	6	245
Spiromesifen	C23H30O4	12	6	169
Isofenphos-methyl	C14H22NO4PS	17	9	189
Prometon	C10H19N5O	24	5	278
Tepraloxydim	C17H24ClNO4	10	3	183
Alanycarb	C17H25N3O4S2	22	7	157
Fenhexamid	C14H17Cl2NO2	14	5	208
Coumaphos	C14H16ClO5PS	19	7	173
Diflufenican	C19H11F5N2O2	26	6	159
Mepanipyrim	C14H13N3	12	5	280
Phenmediphan	C16H16N2O4	29	6	208
Rotenone	C23H22O6	21	5	159
Malathion	C10H19O6PS2	20	7	189
Bupirimate	C13H24N4O3S	13	7	198
Hexaflumuron	C16H8Cl2F6N2O3	18	6	136
Mandipropamid	C23H22ClNO4	19	7	152

Benzoximate	C18H18ClNO5	15	2	172
Dursban	C9H11Cl3NO3PS	14	6	179
Mandipropamid	C23H22ClNO4	17	5	152
Picoxystrobin	C18H16F3NO4	19	6	170
Proquinazid	C14H17IN2O2	10	7	168
Benzoximate	C18H18ClNO5	23	2	172
Boscalid	C18H12Cl2N2O	11	7	183
Picoxystrobin	C18H16F3NO4	13	3	170
Spirodiclofen	C21H24Cl2O4	11	6	152
Ethofumesate	C13H18O5S	27	3	218
Mecarbam	C10H20NO5PS2	26	6	190
Pyraclostrobin	C19H18ClN3O4	12	6	161
Temephos	C16H20O6P2S3	18	6	134
Mixture of Avermectin B1a and B1b	C48H72O14	17	2	72
Azamethiphos	C9H10ClN2O5PS	18	8	193
Carbaryl	C12H11NO2	10	4	311
Carbendazim	C9H9N3O2	34	7	327
Ethyoxyquin	C14H19NO	18	2	288
Mexacarbate	C12H18N2O2	23	6	281
Pirimicarb	C11H18N4O2	18	5	262
Moxidectin	C37H53NO8	15	3	98
Trifloxystrobin	C20H19F3N2O4	14	7	153
Pyripoxyfen	C20H19NO3	23	4	195
Quinalfos	C12H15N2O3PS	15	8	210
Silthiofam	C13H21NOSSi	15	7	234
Difenoconazole	C19H17Cl2N3O3	52	7	154

Fipronil	C12H4Cl2F6N4OS	15	6	143
Cyazofamid	C13H13ClN4O2S	14	3	193
Phenthoate	C12H17O4PS2	15	9	195
Ethofumesate	C13H18O5S	14	2	218
Fenpyroximate	C24H27N3O4	11	7	148
Tolylfluanid	C10H13Cl2FN2O2S2	37	2	181
Carfentrazone-ethyl	C15H14Cl2F3N3O3	17	8	152
Tolclosof methyl	C9H11Cl2O3PS	21	6	208
Carfentrazone-ethyl	C15H14Cl2F3N3O3	37	3	152
Fenazaquin	C20H22N2O	21	4	204
Indoxacarb	C22H17ClF3N3O7	19	7	119
Kresoxim-methyl	C18H19NO4	21	5	200
Indoxacarb	C22H17ClF3N3O7	33	5	119
Kresoxim-methyl	C18H19NO4	40	3	200
Pyridaben	C19H25ClN2OS	15	3	172
Profenofos	C11H15BrClO3PS	13	6	168
Triflumuron	C15H10ClF3N2O3	11	7	175
Flumetsulam	C12H9F2N5O2S	26	7	192
Fuberidazole	C11H8N2O	16	7	340
Sulfentrazone	C11H10Cl2F2N4O3S	13	5	162
Thiabendazole	C10H7N3S	19	6	311
Tebufanpyrad	C18H24ClN3O	15	2	188
Pirmiphos-methyl	C11H20N3O3PS	16	7	205
Picolinafen	C19H12F4N2O2	19	8	166
Quinoxyfen	C15H8Cl2FNO	27	7	204
Teflubenzuron	C14H6Cl2F4N2O2	12	6	164

Oxadiazon	C15H18Cl2N2O3	17	7	182
Metrafenone	C19H21BrO5	25	5	153
Pendimethalin (Penoxalin)	C13H19N3O4	14	6	222
Propaquizafop	C22H22ClN3O5	15	5	141
Benfuracarb	C20H30N2O5S	23	1	152
Benfuracarb	C20H30N2O5S	11	3	152
Fosthiazate	C9H18NO3PS2	15	6	221
Metamitron	C10H10N4O	16	3	309
Phospamidon (Mix of isomers)	C10H19ClNO5P	15	7	209
Sulfentrazone	C11H10Cl2F2N4O3S	11	7	162
Thiametoxam	C8H10ClN5O3S	24	6	215
Flufenoxuron	C21H11ClF6N2O3	12	8	128
Hexythiazox	C17H21ClN2O2S	12	6	178
Carbofuran	C12H15NO3	15	4	283
Chloridazon	C10H8ClN3O	48	8	283
Fosthiazate	C9H18NO3PS2	23	7	221
Imidacloprid	C9H10ClN5O2	16	6	245
Mesosulfuron-methyl	C17H21N5O9S2	14	6	124
Triticonazole	C17H20ClN3O	32	5	197
Azaconazole	C12H11Cl2N3O2	10	7	209
Cymoxanil	C7H10N4O3	23	2	316
Fenarimol	C17H12Cl2N2O	18	6	189
Fluometuron	C10H11F3N2O	13	6	269
Nitenpyram	C11H15ClN4O2	11	5	231
Cymiazol hydrochloride	C12H14N2S	12	7	287
Ethirimol	C11H19N3O	12	7	299

Flumioxazin	C19H15FN2O4	13	2	177
Fluquinconazole	C16H8Cl2FN5O	14	7	167
Flutriafol	C16H13F2N3O	19	6	208
Diethofencarb	C14H21NO4	14	7	234
Dimethachlor	C13H18ClNO2	36	7	245
Ethoprop	C8H19O2PS2	18	6	258
Foramsulfron	C17H20N6O7S	17	6	138
Metobromuron	C9H11BrN2O2	16	6	242
Chlorantraniliprole	C18H14BrCl2N5O2	11	7	130
Guthion	C10H12N3O3PS2	11	6	197
Methabenzthiazurone	C10H11N3OS	10	7	283
Oxadixyl	C14H18N2O4	13	4	225
Quinoclamine	C10H6ClNO2	35	2	302
Spirotetramat	C21H27NO5	14	7	167
Tebuconazole	C16H22ClN3O	16	6	203
Tebuthiuron	C9H16N4OS	12	6	274

Quantification behavior at higher collision energy ranges

All results for pesticides measured at a collision energy range from 50 to 80 eV were filtered using a 5% FDR threshold. At half of the dilution series (1:1,024) 58 metabolites were detected (Supplementary Fig. 8a). The difference in mean standard deviation regarding the theoretical concentration of the automatic and manual analysis is shown in Supplementary Fig. 8b. The median coefficient of variation (CV) of quantified signals was below 20% in all technical replicates (Supplementary Fig. 8c).

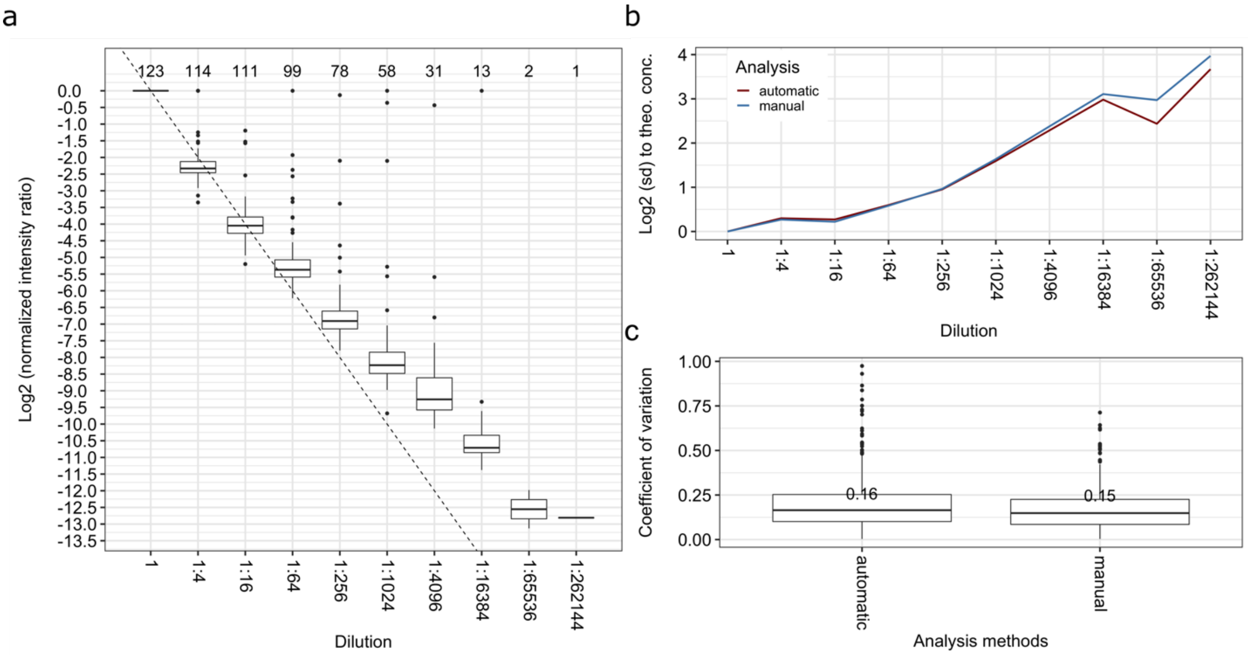

Supplementary Figure 8 | Quantification behavior (CE 50-80 eV). a) Normalized intensity ratio over the dilution series of data acquired with a collision energy of 50-80 eV (normalized for each metabolite adduct combination by the intensity of their highest concentration). The dashed line shows the ideal values (fourfold difference to the next dilution). The number on top is the number of metabolites found in the specific dilution at a 5% FDR cutoff. At half of the dilution series (1:1,024) we could detect 58 metabolites. b) Difference in mean standard deviation regarding the theoretical concentration of the automatic and manual analysis c) Shows the median coefficient of variation (CV across three technical replicates for the automatic and manual analysis (CV < 20%)). For a and b, only metabolites detected in triplicates and below a 5% FDR threshold were analysed and only true positives were considered in the case of panel c. The box plots in a and c indicate median, 25th and 75th percentiles (middle line, Q1 and Q3 within the box respectively), including 1.5x interquartile range whiskers and outliers (single points outside this range).

Comparison of identification performance between DIAMetAlyzer and MS-DIAL

We performed a comparison with MS-DIAL (v. 4.60) based on our ground truth pesticide mix dataset. Here, our constructed assay library was used for the identification of deconvoluted spectra in MS-DIAL. First, the assay library was converted to a spectral library (.msp) using an in house script (https://github.com/oliveralka/MetaboAssayLibToMSPConversion). Secondly, the PesticideMix SWATH files (MTBLS 1108) were converted to .abf using the ABF Converter (v. 1.0 https://www.reifycs.com/AbfConverter/index.html). We then used the sample with the highest concentration of the pesticide mix for the comparison. Data was processed in MS-DIAL using default parameters, additionally allowing retention time for scoring and the adducts [M+H]+, [M+Na]+ and [M+K]+. The spectral library and the experiment file including the SWATH windows were specified (assaylib_20-50_100_ms_dial.msp; Experiment_file.txt). The MS-DIAL results were preprocessed by filtering for compounds identified via spectral library search based on a reference MS2 and by conversion of the retention time to seconds instead of minutes (convert_and_filter_peak_list.py). Then the results were compared to the ground truth dataset and DIAMetAlyzer results at 5% FDR (comparison_ground_truth_pyprophet_ms-dial.py). The comparison was visualized using an R script (vis_comp_MS-DIAL.Rmd). DIAMetAlyzer

was able to identify 156 true positives and 3 false positive compounds in comparison to the ground truth. MS-DIAL with an identification threshold of 0.8 and retention time scoring enabled, was able to identify 84 true positives, 5 false positives and was not able to identify 70 compounds (false negatives) (Supplementary Fig. 9). Here, we could show the advantage of DIAMetAylzer targeted extraction strategy with false-discovery rate control based on reference compounds in comparison to non-targeted deconvolution. We would like to state that the functionality of MS-DIAL is focused on the identification of unknown compounds and is dependent on the spectral library space given to the software.

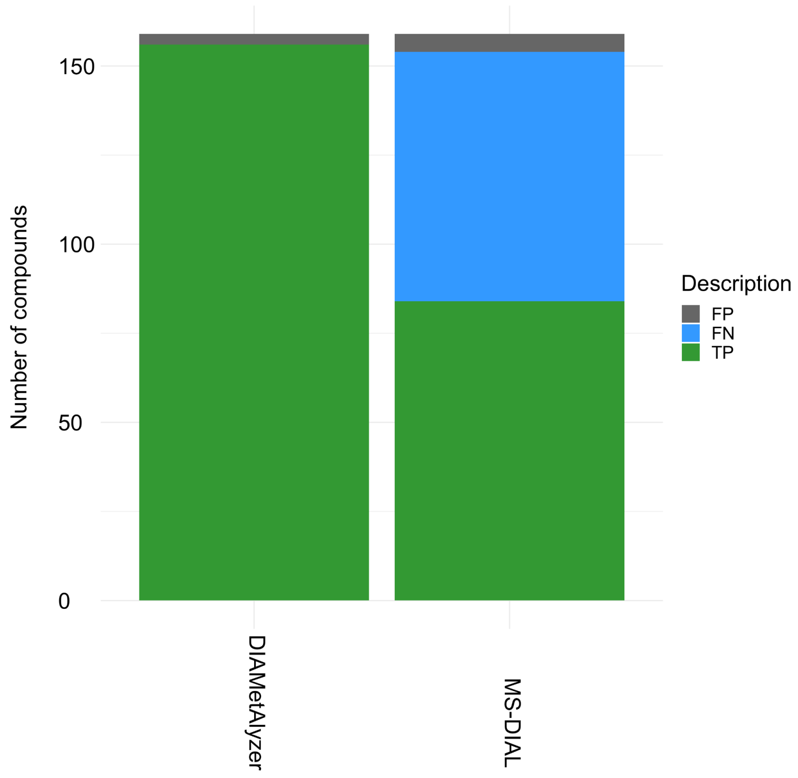

Supplementary Figure 9 | Identification performance of DIAMetAlyzer in comparison with MS-DIAL based on the generated assay library. DIAMetAlyzer was able to identify 156 true positive (TP) and 3 false positive (FP) compounds in comparison to the ground truth. MS-DIAL with an identification threshold 0.8 (default) and retention time scoring enabled, was able to identify 84 true positives, 5 false positives and was not able to identify 70 compounds (FN: false negatives).

Additional methods for the comparison with MetaboDIA using the AMD dataset

Raw data of MTBLS417 (https://www.ebi.ac.uk/metabolights/MTBLS417) was provided by the authors and data was processed as previously described[14]. DDA data was centroided using the qtofpeakpicker and msconvert for conversion into mzML/mzXML. DIA data conversion was performed using msconvert. MetaboDIA (Version 1.3) processes DDA and DIA data in centroided mode (mzXML), in contrast to DIAMetAlyzer which uses centroided DDA (mzML) and profile DIA (mzML) data. An accurate mass search database consisting of HMDB (4.0) and LIPIDMAPS (092020) was constructed, with the entries from the LIPIDMAPS structure file converted into a readable format (Lipid_Enrichment_prepare_AMS.knwf).

Afterwards, duplicates were filtered from the database (reoderDuplicatesMapping.py), and the HMDB and LIPIDMAPS entries were combined (AppendIdentifiersFromOtherDB.py). For MetaboDIA both databases were combined in a Database.txt file. MetaboDIA was used for the analysis and in case of DIAMetAlyzer, an OpenMS development version (14f627e) was used with support for SIRIUS 4.5, allowing internal decoy generation and feature linking for targeted and untargeted experiments using the AssayGeneratorMetabo library generation. For MetaboDIA, the DIA data needed to be processed with DIA-Umpire[15] (Version 2.1.6) (diaumpire_slurm_mtbls417.sh, params_diaumpire_mtbls417.se_params). DDA and DIA data were processed with XCMS[16] and CAMERA[17] for feature detection and identification. The DDA/DIA workflow from MetaboDIA was run, by specifying the related files, database and adducts (metaboDIA_run.Rmd). Feature detection, adduct grouping and precursor correction for DIAMetAlyzer was performed using KNIME (20200922_processing_MetaboDIA_FFM_MAD_HRPMC.knwf) followed by accurate mass search (AccurateMassSearch.sh). To decrease the runtime of data processing, the assay generation and targeted extraction step was performed on a cluster infrastructure. The library was constructed by either using prior MS1 identification (agm_mt1_02.sh) or with the addition of non-identified features (agm_mt1_unknown_02.sh). The spectral library stemming from MetaboDIA was converted into an assay library (convertSpetralLibrarytoAssayLibrary_1.4.py) and decoys were generated using the DecoyGeneratorMetabo fragdb method (generateMultiDecoys.sh). A combined library was constructed using the DIAMetAlyzer and the converted MetaboDIA library (construct_combined_library.ipynb). All libraries were used for targeted extraction similarly (osw_mt1_67_02.sh). Furthermore, statistical validation was performed using PyProphet (merge_score_export_pyprophet.sh). Results were then processed by DIAlignR[18], which was extended to allow for metabolomics data processing, to reassess values based on chromatogram retention time alignment (DIAlignR.Rmd). In the following, features with a FDR below 0.05 and peak group level 1 were used for post-processing analysis, which includes library comparison based on the molecular formula, adduct and retention time of a feature (comparison_lib.ipynb). Data was quantitatively compared based on molecular formula and adduct (compare_lib_and_quant.ipynb), with a further comparison of the quantification matrix between features found in MetaboDIA and DIAMetAlyzer to assess the difference of non-targeted vs targeted extraction/quantification (compare_analysis.ipynb). Assessment of differences between the groups was performed using limma with Benjamini & Hochberg correction for multiple testing (quant_analysis.Rmd).

Combining assay libraries

The created AssayGeneratorMetabo Node in our DIAMetAlyzer pipeline provides a robust, reproducible, automated method to build the target-decoy assay library in the OpenMS ecosystem using the fragmentation tree re-rooting method[3].

The combination of spectral/assay library information of other tools is not straightforward due to interoperability issues between the tools and their used data format.

In most cases individual solutions need to be provided/developed to convert the original format to the OpenSWATH assay library format. In the case of MetaboDIA we provide a script for the conversion (convertSpetralLibrarytoAssayLibrary_1.4.py).
The fragmentation re-rooting method (SIRIUS) cannot be performed since the MS1 and MS2 spectra information needed is not available on assay library level. For that purpose, we investigated decoy generation methods on the assay library level, which are easier to use and still perform reasonably well. Here, we provide the DecoyGeneratorMetaboTool (https://github.com/oliveralka/DIAMetAlyzer_additional_code/blob/master/additional_tests_decoy_methods/TOOL/DecoyGeneratorMetaboTool_2.0.py)[19]. For further details regarding the decoy methods on library level please see Supplementary Fig. 18, 19. The so generated target and decoy assay library can then be appended to the one from the AssayGeneratorMetabo node. The combined library can be used in the DIAMetAlyzer workflow for the DIA data analysis.

Library comparison (MetaboDIA vs DIAMetAlyzer)

We compared the different libraries and their entries based on molecular formula, adduct and retention time information (Supplementary Fig. 10). MetaboDIA produces the smallest library, followed by the native DIAMetAlyzer. Combining both libraries leads to an increase in the number of features and stays on the conservative side. Allowing features without MS1 identification in addition to the native DIAMetAlyzer library leads to a further increase of features in the library. Here, SIRIUS is used for the assessment of the molecular formula and the fragment annotation internally.

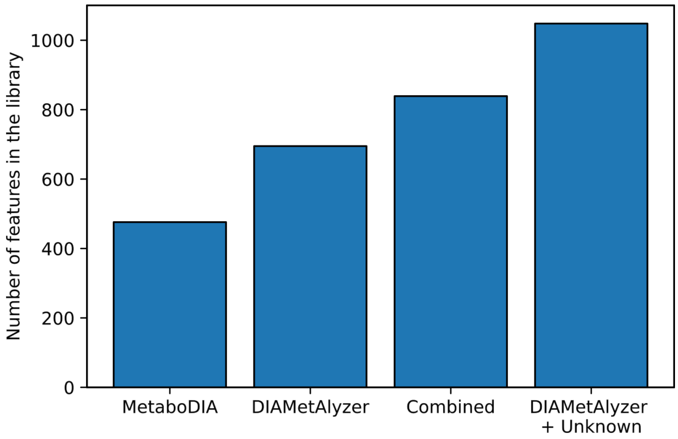

Supplementary Figure 10 | Library comparison MetaboDIA vs DIAMetAlyzer. Comparison of libraries and their entries based on molecular formula, adduct and retention time information. MetaboDIA produces the smallest library (n = 440), followed by native DIAMetAlyzer (n = 695). Combining both libraries leads to an increase and stays on the conservative side (n = 839). The option which presents the highest number of entries in the library is to allow features, without an MS1 identification and use SIRIUS to assess the molecular formula and fragment annotation (n = 1048).

The difference in DDA feature detection and linking of MetaboDIA and DIAMetAlyzer

We are using the DDA data to generate our targeted assay library. Two important steps in this process are feature detection and feature linking. We used the OpenMS feature detection algorithm (FeatureFinderMetabo) developed by Kenar *et al.*, 2014[20]. A short description of the differences of the feature detection algorithms (XCMS[16,17], MzMine[21], Maven[22]) can be found in the publication. In short, FeatureFinderMetabo was compared to XCMS using a simulated ground truth dataset. Here, a similar feature overlap of around 66% was reported, as well as, a higher recall, precision and F-score for the FeatureFinderMetabo.

For the feature detection and linking comparison of MetaboDIA and DIAMetAlyzer, we used detected features with a minimum of one mass trace (monoisotopic peak) and linked in at least 50% of the samples. Based on the assay library comparison, DIAMetAlyzer and MetaboDIA overlap in 113 features, whereas MetaboDIA (XCMS/CAMERA) and DIAMetAlyzer (FeatureFinderMetabo) found 39 and 158 exclusive features respectively. To compare the feature detection and linking based on the library comparison, overlapping and non-overlapping features were exported to an excel sheet, manually converted to edta and automatically converted to featureXML. The featureXML was then used for visualization in OpenMS TOPPView. Two DDA files were chosen randomly (one of each sample group: PH697172_pos_IDA-PH697172; CS56422_pos_IDA-CS56422) and the features were manually inspected. Comparing the feature detection and feature linking results of the non-overlapping features in more detail, only 8 of the 39 features in the MetaboDIA library have not been detected by DIAMetAlyzer (FeatureFinderMetabo). The other 31 features are filtered in the assay library generation, discussed in more detail below.

In terms of feature detection XCMS/CAMERA, were in some cases not able to separate features (Supplementary Fig 11a), but were able to better detect features with a tailing mass trace (Supplementary Fig 11b) or features that start with a maximum (e.g. at the beginning of the retention time gradient) (Supplementary Fig. 11c,d).

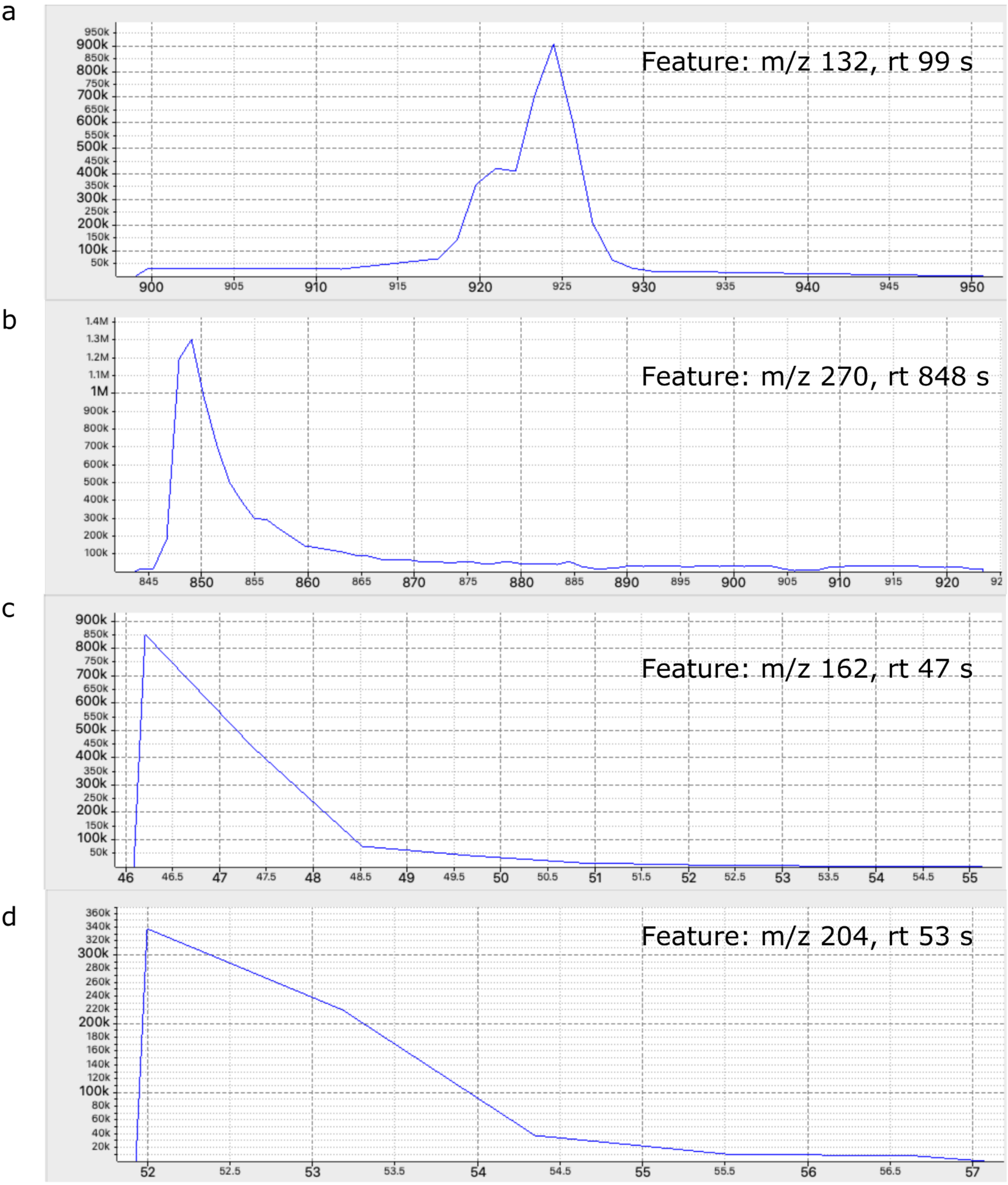

Supplementary Figure 11 | Examples for exclusive features of MetaboDIA DDA feature detection via XCMS/CAMERA. a) Overlapping features were detected as one feature (m/z 132, rt 99 s). b) Features with tailing mass traces were detected more frequently (m/z 270, rt 99 s). c,d) Features starting at a maximum in the beginning of the retention time gradient were detected more often (m/z 162, rt 47 s; m/z 204, rt 53 s). Mass-to-charge (m/z) and retention time (rt) in seconds (s) are associated with the features based on the consensus found over multiple samples.

The DIAMetAlyzer (FeatureFinderMetabo) picks up low intensity features more consistently (Supplementary Fig. 12a-c). In addition, the manual inspection suggests that the FeatureFinderMetabo was able to detect and split multiple features "hidden" in longer mass traces (Supplementary Fig. 12d.)

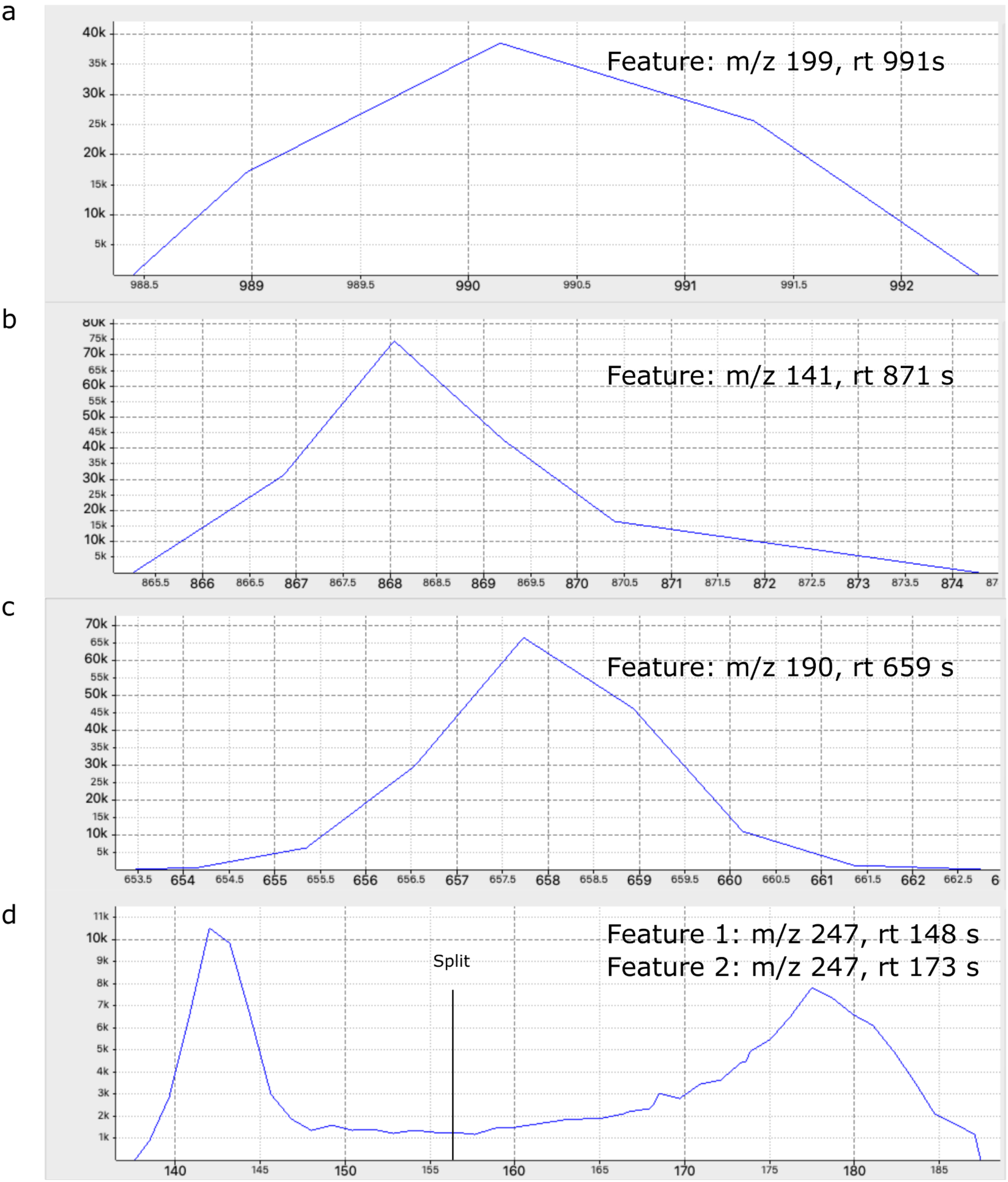

Supplementary Figure 12 | Examples for exclusive features of DIAMetAlyzer DDA feature detection via FeatureFinderMetabo. a,b,c) Low intensity features with an intensity of 40,000 – 70,000 were detected more consistently (m/z 199, rt 991 s; m/z 141, rt 871 s; m/z 190, rt 659 s). d) FeatureFinderMetabo was able to detect and split features together in a longer mass trace (m/z 247, rt 173 s & m/z 247, rt 173 s). Mass-to-charge (m/z) and retention time (rt) in seconds (s) are associated with the features based on the consensus found over multiple samples.

In general, we think a more refined parameter optimization of both algorithms would still be able to detect additional features in this dataset.

The presented features in Figures 11 and 12 do not represent the main population of features detected in the analysis. In case of Figure 11 c,d, the analytes are eluting in the beginning of the gradient and are somewhat cut-off but are still features which can be detected with the

XCMS algorithms. In the case of Figure 12 a-c, the feature intensity span in the file was roughly from 7,523 to 72,903,272. All features shown have a maximum intensity of 75,000 and can be deemed low intensity.

The features presented in Figures 11 and 12 do not show an optimal chromatographic shape with around 6-8 peaks. Supplementary Fig. 13 shows the chromatograms two mid-range intensity features and one high intensity feature as representatives of the main feature population in the dataset. Please be aware that due to different axis ranges in intensity and retention time, the figures are not directly comparable, but should give an indication of the feature population in the sample.

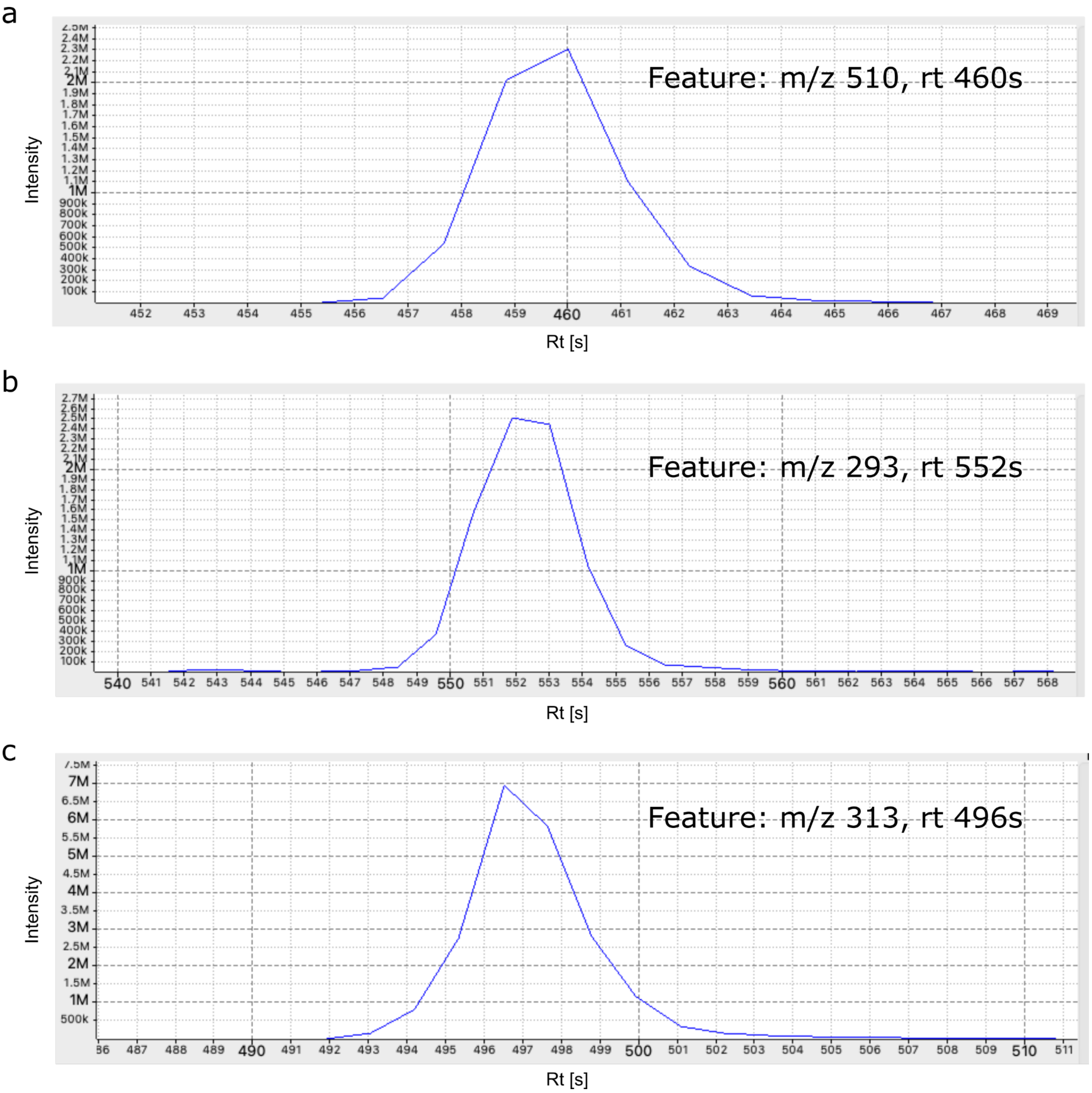

Supplementary Figure 13 | Examples for mid and high intensity features detected in the DDA data. a,b) Mid intensity features with an intensity of around 2,000,000 were detected in both algorithms. c) High intensity feature with an intensity of 7,000,000. All three features were extracted from DDA data and are shown as representation of the chromatographic outline of the main feature population in the dataset.

A big difference between the tools is introduced in the feature linking/assay library generation step. MetaboDIA detects and links features independent of their identification. In DIAMetAlyzer the feature linking is performed after fragment annotation. Here, features with less than four fragment peaks in the MS2 spectra are filtered out by SIRIUS. Further filtering depends on the parameterisation of the workflow. Using solely known compounds, features without identification or without a correct fragmentation annotation are filtered out before the linking process. This leads to the filtering of linked features based on occurrence of their identification. For example, if a feature was linked in 67 samples, but was only correctly identified in 13 samples, the feature in case of the DIAMetAlyzer is filtered, since it is not available with a valid identification in 50% of the samples. This step is performed to ensure a high-quality assay library based on identification and fragment annotation.

In conclusion, the feature detection in DIAMetAlyzer can detect more features, but the linking is more stringent, dependent on the application, to ensure a high-quality assay library. One of the strong points of DIAMetAlyzer is that it allows for the aggregation of information in the assay library e.g. based on the findings of other tools or spectral libraries.

In terms of stability and reproducibility, since feature detection, adduct grouping and identification are distinct steps to construct the assay library, a change in parameters may lead to differences in the results. It is advisable to optimize the feature detection parameters to the specific dataset, to improve the feature coverage. The changing of parameters should not impair the general robustness of the workflow. In terms of reproducibility, KNIME allows to save and export the workflow with the adjusted parameters to fit the experimental setting. We would advise supplying the specific workflow or script which was used for the analysis. This will allow for reproducibility independent of the parameter changes.

Targeted vs non-targeted quantification (MetaboDIA vs DIAMetAlyzer)

Based on the set of molecular formulas and adducts, MetaboDIA was able to quantify 54 features in comparison to DIAMetAlyzer, which was able to quantify 440 features using the same library (MetaboDIA) (Supplementary Fig. 14a). This discrepancy was discussed with the developers of MetaboDIA. They stated that MetaboDIA is not in active development anymore. Due to its dependence on XMCS, CAMERA and DIA-Umpire, changes in one of these software suites may lead to such performance issues. Based on the quantified overlapping features the targeted extraction method used in DIAMetAlyzer has a clear advantage over the method used by MetaboDIA and is in most cases able to quantify a compound in every sample (Supplementary Fig. 14b).

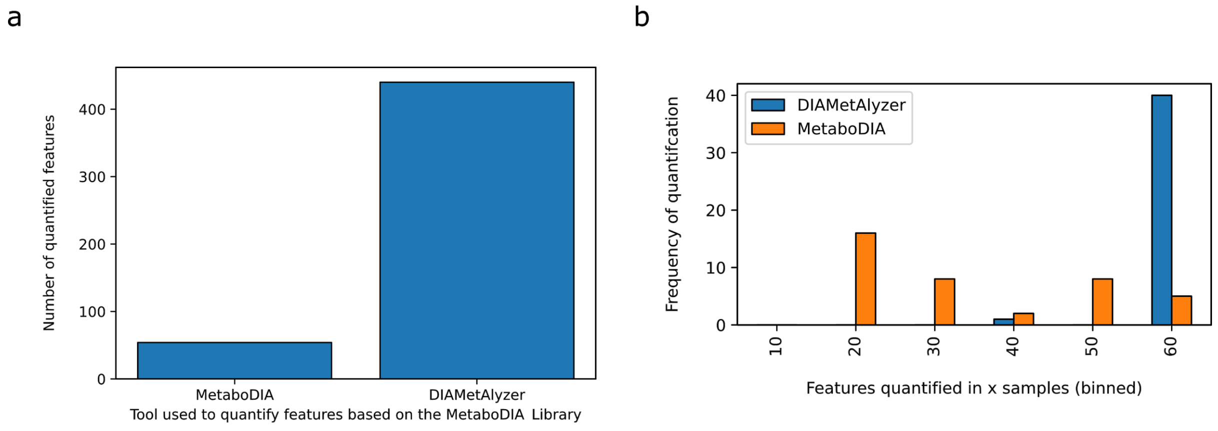

Supplementary Figure 14 | Quantification comparison between MetaboDIA and DIAMetAlyzer. a) Based on the set of molecular formula and adduct MetaboDIA was able to quantify 54 features in comparison DIAMetAlyzer was able to quantify 440 features using the same library (MetaboDIA) b) Shows that most features, which were quantified by both tools, have quantitative values in 50-60 samples when DIAMetAlyer is used. In contrast, a higher variation in this frequency is seen for MetaboDIA.

Patient group comparison of the AMD data

DIAMetAlyzer + Unknown analysis was used to perform a principal component analysis to assess the class separation based on the determined features. Using all available features (Supplementary Fig. 15) or features that showed a significant difference between groups (5% FDR). In both cases the control shows a distinct group but cannot be separated by PC2. The separation in PC1 direction leads to the conclusion of a batch effect since a few samples from each group (CNV, PCV) are separated by PC1. This occurrence does not arise from the extraction batch or injection order. Unfortunately, not enough metadata is present to explain this effect.

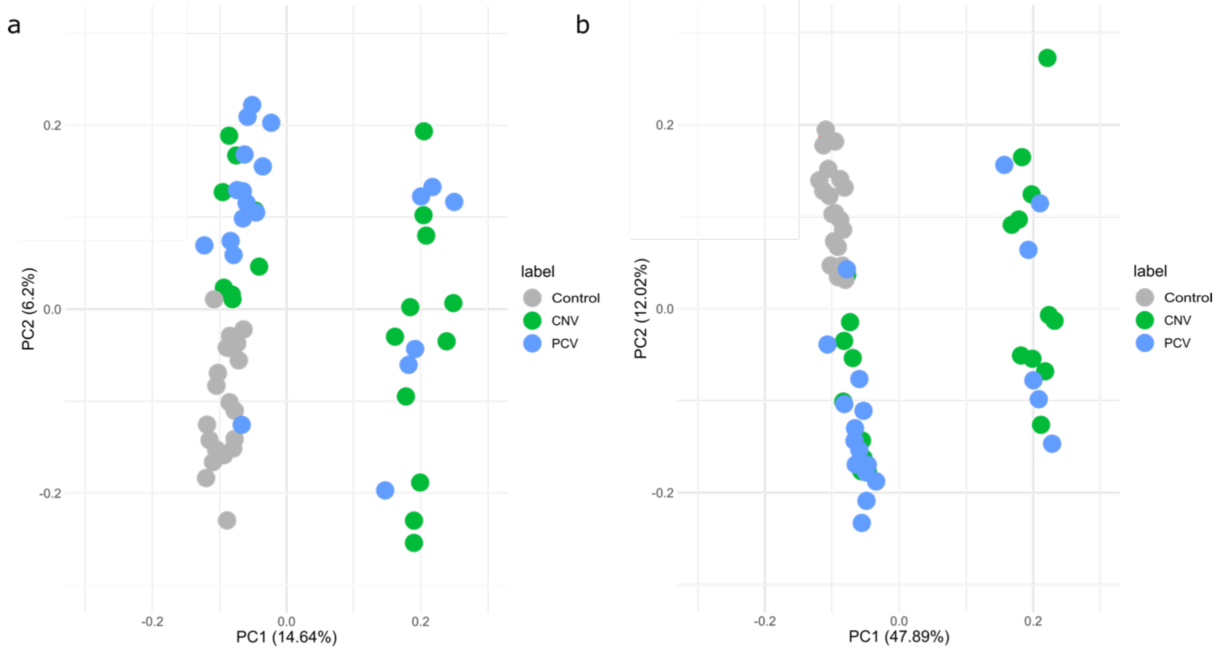

Supplementary Figure 15 | Principal Component Analysis (PCA) to assess group separation based on features. a) PCA based on all available features b) Based on features which showed a significant difference between groups (5% FDR). In both cases, the control can almost be separated by PC2.

Differential expression analysis of AMD data

Differential expression analysis was performed with limma to assess the differences between the control, CNV and PCV groups. In the case of control vs CNV 208 analytes show a significant difference between the groups. 79 analytes in case of control vs PCV. 49 of these analytes are found to be differentially regulated in both groups. In the comparison of CNV and PCV, no significant differentially regulated analytes could be detected. The compound classes of the differentially regulated analytes were retrieved using MetaboAnalyst[23] (Version 5.0) - over representation analysis - based on the first compound name annotated by accurate mass search. Here, the super-, main- and sub-classes were identified for metabolites and lipids. CNV and PCV in comparison to the control group based on the differential expression analysis via limma, consist of metabolites from compound classes such as glycerophospholipids, organic heterocyclic compounds, sterol lipids, fatty acids, amino acids and dipeptides.

Quantification and evaluation of AMD biomarkers and additional candidates

Carnitines and their metabolites are mainly involved in fatty acid metabolism. Oleoylcarnitine ($C_{25}H_{47}NO_4$; P_{CNV}=0.002; P_{PCV}=0.01), as well as L-Palmitoylcarnitine ($C_{23}H_{45}NO_4$; P_{PCV}=0.02), are upregulated by around 1.5 times in contrast to the control in both or PCV, respectively (Supplementary Fig. 16a,b). These findings are following the research of an altered carnitine shuttle pathway in macular degeneration[24]. Our findings suggest that Linoelaidylcarnitine ($C_{25}H_{45}NO_4$; P_{CNV}=0.04; P_{PCV}=0.03) which showed a similar increase in addition to the others might be a potential biomarker for AMD (Supplementary Fig. 16c). Further possible biomarkers associated with AMD were determined from serum in previous studies, such as phenylalanine, hypoxanthine, tyrosine[25]. We found hypoxanthine levels were significantly increased in CNV ($C_5H_4N_4O$; P_{CNV}=0.006) by 3.9 times in contrast to the control, which affects the purine nucleotide cycle and can lead to apoptosis of photoreceptors[26,27](Supplementary Fig. 16d). In addition, gamma-Glutamylphenylalanine ($C_{14}H_{18}N_2O_5$; P_{CNV}=0.002; P_{PCV}=0.0006) and gamma-Glutamylisoleucine ($C_{11}H_{20}N_2O_5$; P_{CNV}=0.01; P_{PCV}=0.04) were deregulated in both patient groups (Supplementary Fig. 16e,f). Increased serum gamma-glutamyl transferase (GGT) levels were reported as risk factors for AMD[28]. This suggests that gamma-Glutamylphenylalanine with higher intensity by around 1.6 times in contrast to the control and gamma-Glutamylisoleucine (1.7 times increase) could be used as metabolic markers in this case.

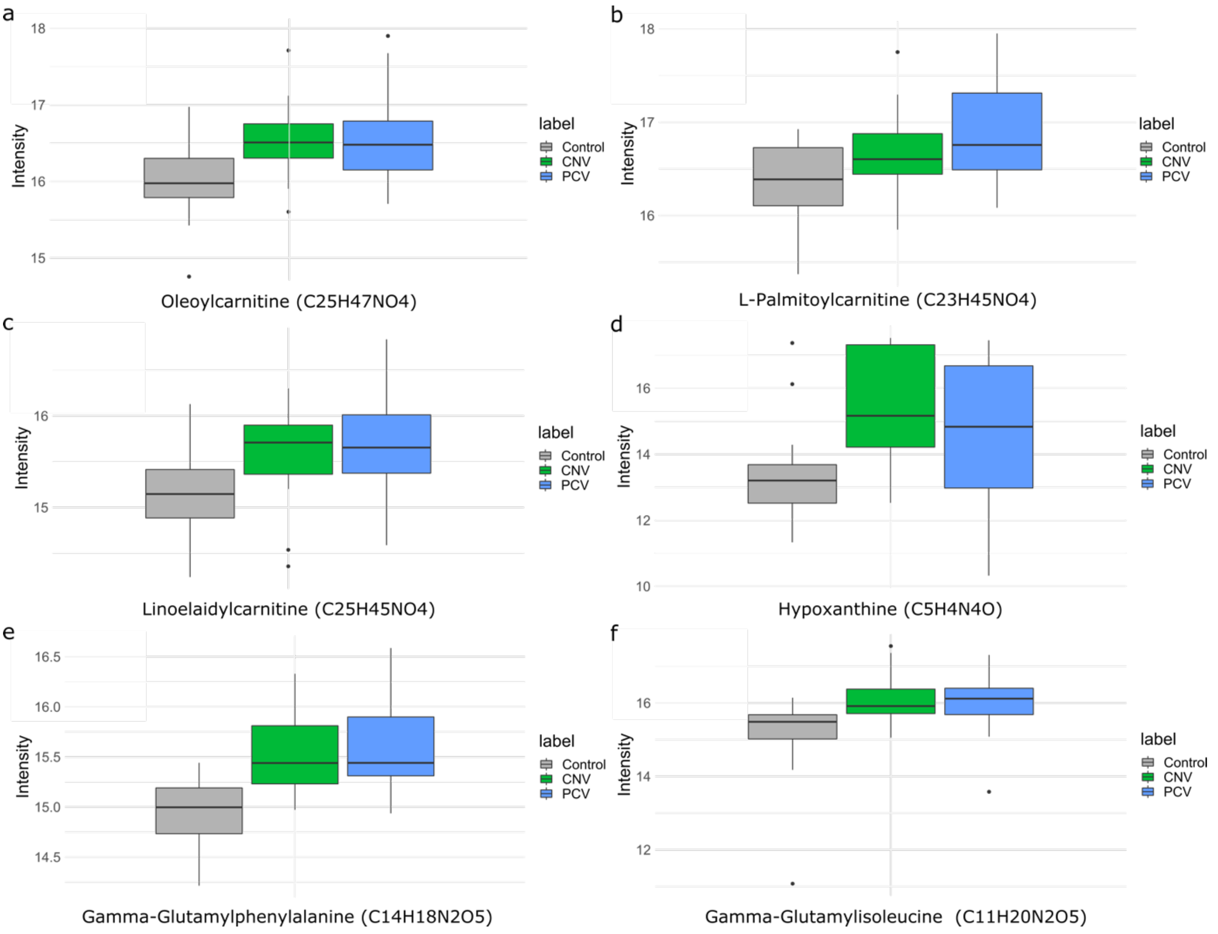

Supplementary Figure 16 | Quantification of biomarkers and additional candidates I. a) Oleoylcarnitine (C25H47NO4; P_{CNV}=0.002; P_{PCV}=0.01) is upregulated by around 1.5 times in both patient groups in contrast to the control group. b) L-Palmitoylcarnitine (C23H45NO4; P_{PCV}=0.02) show the same increase in the case of PCV. c) Linoelaidylcarnitine (C25H45NO4; P_{CNV}=0.04; P_{PCV}=0.03) showed a similar increase and might be a potential biomarker for AMD. d) Hypoxanthine levels were significantly increased in CNV (C5H4N4O; P_{CNV}=0.006) by 3.9 times in contrast to the control. e) gamma-Glutamylphenylalanine (C14H18N2O5; P_{CNV}=0.002; P_{PCV}=0.0006) with an increase of 1.6 times in contrast to the control. f) gamma-Glutamylisoleucine (C11H20N2O5; P_{CNV}=0.01; P_{PCV}=0.04) showed a 1.7 increase in comparison to the mean intensity of the control. a-f) The identification of the compounds is based on MS1 accurate mass search and MS2 fragment annotation. Differential expression was assessed using limma with Benjamini-Hochberg correction. Box plots indicate median, 25th and 75th percentiles (middle line, Q1 and Q3 within the box respectively), including 1.5x interquartile range whiskers and outliers (single points outside this range). Control: gray, n = 20 biologically independent samples, CNV (choroidal neovascularization): green, n = 20 biologically independent samples, PCV (polypoidal choroidal neovascularization): blue, n = 20 biologically independent samples.

As a validation, dityrosine, which plays a role in oxidative stress and is associated with macular degeneration[29], was increased by around 1.7 to 2.4 times in contrast to the control (C18H20N2O6; P_{CNV}=0.002; P_{PCV}=0.03) (Supplementary Fig. 17a). We found additional candidates without identification at m/z 601.271468 and a retention time 579 s (Unknown A; P_{CNV}=0.0002; P_{PCV}=0.000008) with an intensity increase of 5.2 and 5.7 in comparison to the control and at m/z 944.360964 and 311 s (Unknown B; P_{CNV}=0.005; P_{PCV}=0.03) with an intensity increase of 1.7 and 1.9 respectively (Supplementary Fig. 17b,c). An additional interesting aspect is the significantly deregulated compounds 5,8,11,14-Eicosatetraenoic acid (EPA) (C20H32O2; P_{PCV}=0.01; P_{CNV}=0.04), after removing one outlier in the CNV patient group, and 4,7,10,13,16,19-Docosahexaenoic acid (DHA) (C22H32O2; P_{PCV}=0.006;

P_{CNV}=0.008) with an increase of 1.4 to 2.0 times in contrast to the control, have previously been associated with a reduced risk for neovascular AMD[30] (Supplementary Fig. 17d,e). An explanation for this finding in the patient groups could be an Omega-3 fatty acids rich diet, which is often advised to AMD patients due to their anti-inflammatory properties[31,32]. The identification results of the differential expression analysis are based on putative identifications via MS1 accurate mass search and MS2 fragment annotation, corresponding to a level 3 identification[33]. Here, to reach a level 1 identification, additional experiments in follow up studies are necessary to validate the potential biomarkers.

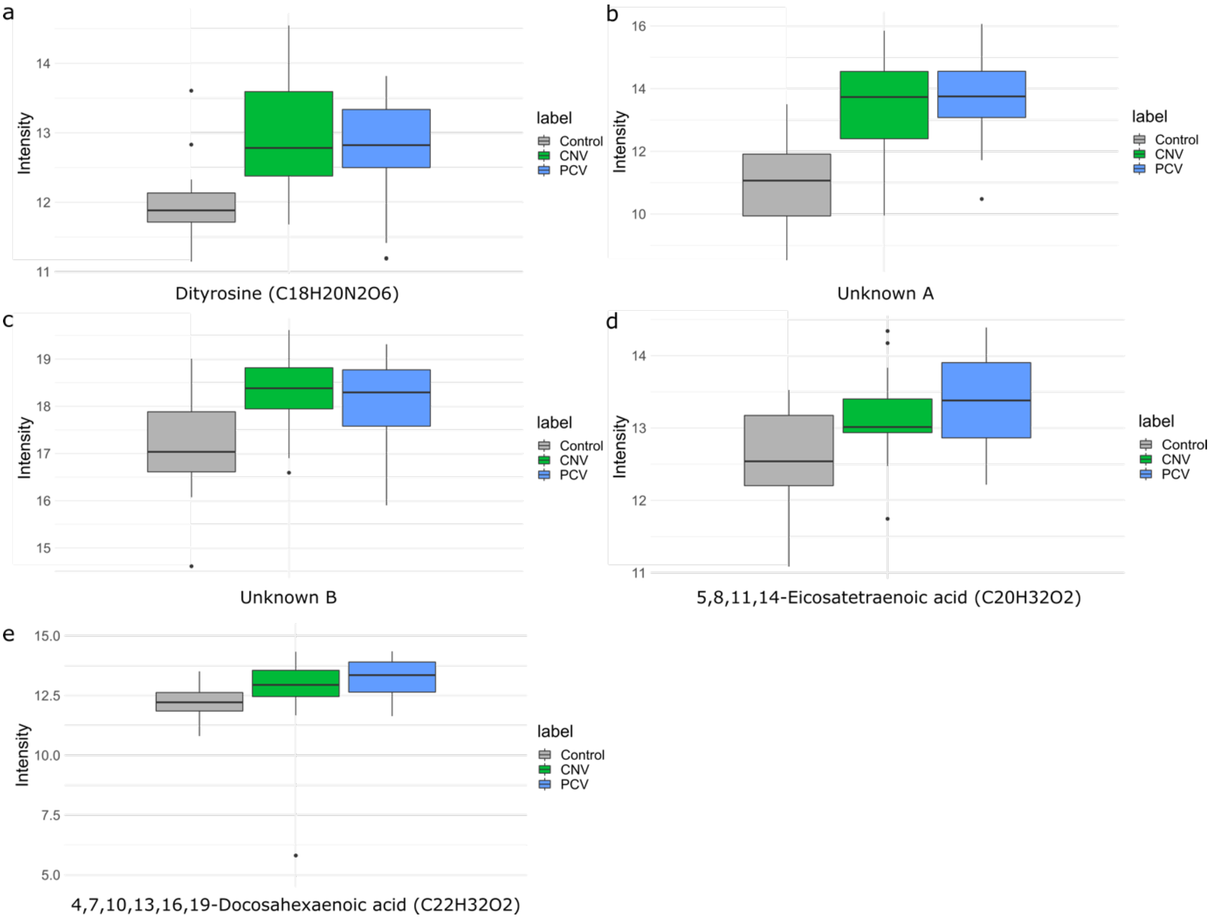

Supplementary Figure 17 | Quantification of biomarkers and additional candidates II. a) Dityrosine, was found to be increased by around 1.7 to 2.4 times in contrast to the control (C18H20N2O6; P_{CNV}=0.002; P_{PCV}=0.03). b) Unknown A at 601.271468 m/z and a retention time 579s (P_{CNV}=0.0002; P_{PCV}=0.000008) with an intensity increase of 5.2 and 5.7 in comparison to the control c) Unknown B at 944.360964 m/z and 311s (P_{CNV}=0.005; P_{PCV}=0.03) with an intensity increase of 1.7 and 1.9 respectively. d) 5,8,11,14-Eicosatetraenoic acid (EPA) (C20H32O2P_{PCV}=0.01; P_{CNV}=0.04) and e) 4,7,10,13,16,19-Docosahexaenoic acid (DHA) (C22H32O2; P_{PCV}=0.006; P_{CNV}=0.008) with an increase of 1.4 to 2.0 times in contrast to the control. a-f) The identification of the compounds is based on MS1 accurate mass search and MS2 fragment annotation. Differential expression was assessed using limma with Benjamini-Hochberg correction. Box plots indicate median, 25th and 75th percentiles (middle line, Q1 and Q3 within the box respectively), including 1.5x interquartile range whiskers and outliers (single points outside this range). Control: gray, n = 20 biologically independent samples, CNV (choroidal neovascularization): green, n = 20 biologically independent samples, PCV (polypoidal choroidal neovascularization): blue, n = 20 biologically independent samples.

Further decoy generation methods and evaluation

Various potential decoy methods were evaluated. Fragdb: This method performs a random sampling of *n* fragments with a lower mass than the current precursor. Linmzperm: Instead of using masses directly this method uses the mass difference between the precursor and fragments. Here, the mass differences were calculated between the precursor and the first fragment, then the first and the second fragment and so on. Afterwards, the mass differences were shuffled randomly and subtracted from the precursor or previous fragment, generating new fragment masses with an in total similar mass difference. Since the last fragment always had the same total mass difference the mass of a $-CH_2$ was added to the new fragment mass. If the same mass difference occurred twice, a $-CH_2$ mass was added to the first fragment mass as well. Rtperm: This method performs a retention time permutation for the precursor and its fragments, within a certain minimal retention time difference. This difference should be higher than the retention time parameter set in OpenSWATH and has to be in the retention time range used in the experiment. The decoy retention time is generated such as decoy_rt = decoy_rt + ([0.0 - 1.0] - 0.5) x 400, where decoy_rt is initialized with the same value as the target retention time. Re-rooting: Here, the fragmentation tree-based method from Passatutto was used. The fragmentation trees were acquired using the fragment annotation via SIRIUS4. The SIRIUS4 format had to be parsed into a Passatutto compatible format. Then the method was called, and decoy spectra were used to extract transitions. In the case of overlapping transition and decoy-transition masses after extraction, a $-CH_2$ mass was added to the overlapping decoy transition. To ensure the same number of targets and decoys, if the re-rooting of the tree failed or the fragments were similar to the target ones, $-CH_2$ was added to the original fragment masses as a fallback mechanism to ensure the generation of a decoy. The fallbacks were used in around 13% and 5% of the cases, respectively. Afterwards, the *n* highest intensity peaks were extracted to use in the target-decoy assay library. The following analysis was performed using the 20-50 eV collision energy data. With the introduced metabolomics score filter in PyProphet all decoy methods perform rather well (Supplementary Fig. 18). The re-rooting method has a conservative tendency (overestimating the FDR slightly). Our aim was to establish a method, which can be used on a multitude of different datasets, in our humble opinion it is better to stay on the conservative side for FDR filtering, especially if the area-under-the-curve for most decoy methods are reported to be in the same range.

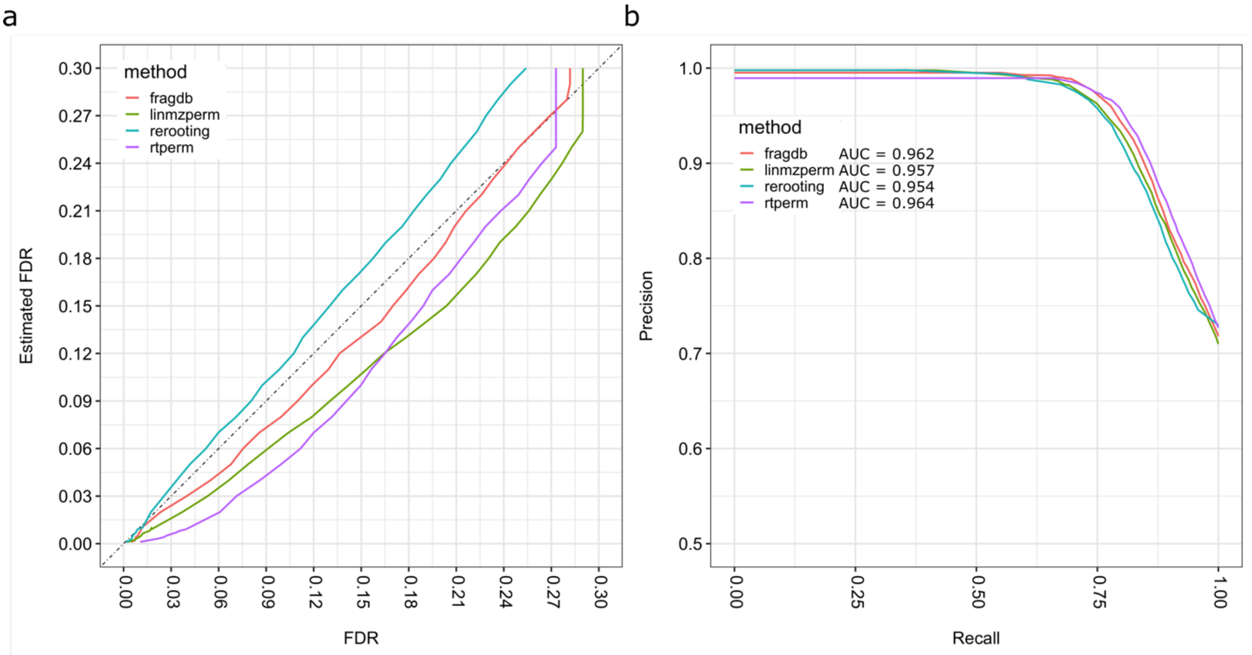

Supplementary Figure 18 | Identification accuracy of DIAMetAlyzer on the pesticide spike-in dataset using different decoy methods. a) The estimated FDR versus the ground truth FDR. The continuous line at 45 degrees shows the optimal values. b) Precision-Recall curve with the area-under-the-curve (AUC (fragdb) = 0.962 , AUC (linmzperm) = 0.957, AUC (rerooting) = 0.954, AUC (rtperm) = 0.964).

In addition, we looked at the impact of adding a -CH_2 mass at different steps in the decoy generation method. Adding a -CH_2 mass is plausible for organic compounds since it can be added to the carbon backbone. There are two situations, where a -CH_2 mass can be added to one or more decoy transitions. First, in the case of overlapping transition and decoy-transition masses after extraction, a -CH_2 mass was added to the overlapping decoy transition (insertion - in). Second, if re-rooting (passatutto) of the fragmentation tree was not possible, -CH_2 mass was added to all the target transitions, which were then used as decoy transitions (shift). These fallbacks were used in 13% and 5% of the cases respectively. Adding a -CH_2 mass in these cases would allow it to have a similar number of targets and decoys, which is not ultimately necessary for the LDA approach used by PyProphet. Here, we tested all combinations of this method. Original: Re-rooting with insertion and the shift. WInwoShift: Re-rooting with insertion without shift. WoInwoShift: Re-rooting without insertion and without shift. WoInwShift: Re-rooting without insertion with shift. In terms of performance, there are no important differences between the described methods, in terms of FDR estimation and precision-recall (Supplementary Fig. 19). A major difference in the disparity between the number of decoys for the specific sets (Number of decoys: original = 165, wInwoShift = 155, woInwoShift = 132, woInwShift = 142). As stated previously a slight discrepancy between the targets and decoys is not problematic for the LDA approach. We decided to further use the re-rooting method with insertion and shift, to allow for a robust method applicable on a lot of different datasets. In our opinion, if the number of decoys is not far below the number of targets the other methods can be used in a similar fashion.

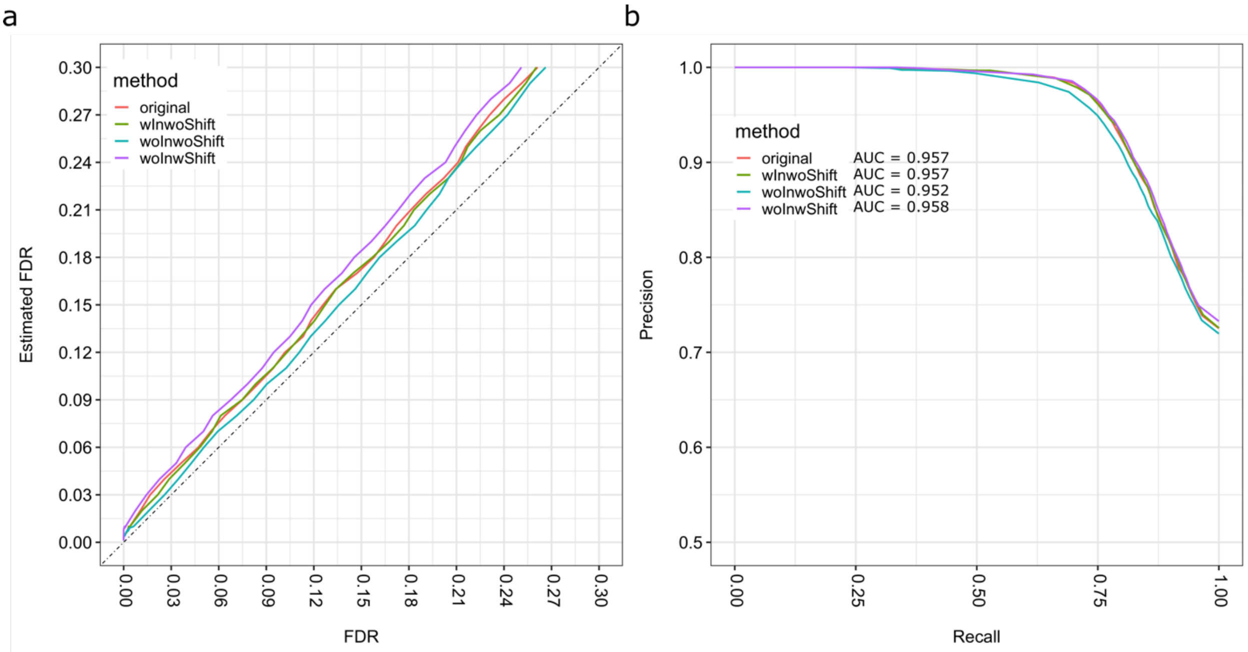

Supplementary Figure 19 | Identification accuracy of the DIAMetAlyzer on the pesticide spike-in dataset using the -CH2 decoy fallback. a) The estimated FDR versus the pseudo ground truth. The continuous line at 45 degrees shows the optimal values. b) Precision-Recall curve with the area-under-the-curve (AUC (original) = 0.957, AUC (wInwoShift) = 0.957, AUC (woInwoShift) = 0.952, AUC (woInwShift) = 0.958).

Comparison of DDA and DIA data

We visually compared the DDA and DIA XICs for the highest concentration and chose two representatives (Supplementary Fig. 20). Both show a similar chromatogram shape on the MS1 level. At the MS2 level, the peak depends on the trigger time of the instrument (the chromatogram shape is based on the interpolation used in the Skyline visualization). For DIA MS2 the chromatogram is clearly visible and co-elutes exactly with the MS1 chromatogram.

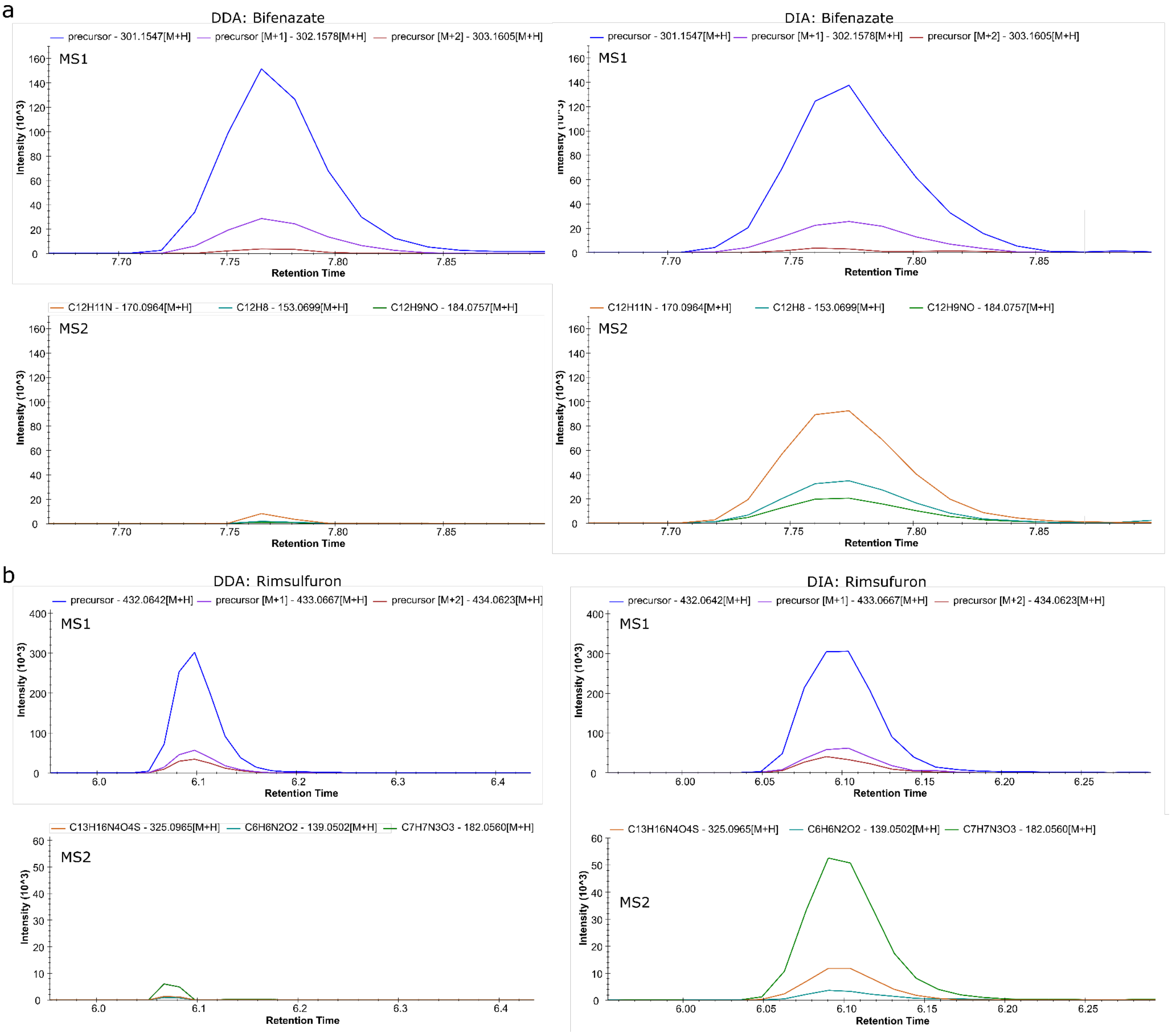

Supplementary Figure. 20 | Examples for MS1 and MS2 XICs from DDA and DIA data. The study data representative pesticides Bifenazate (a) and Rimsulfuron (b) show a similar MS1 XIC in DDA and DIA.

Example workflow

Example workflow for the usage of the DIAMetAlyzer Pipeline in KNIME[34,35] (Supplementary Fig. 21). Inputs are the SWATH-MS data in profile mode (.mzML), a path for saving the new target-decoy assay library, the SIRIUS[36] (Version 4.0.1) executable, the DDA data (.mzML), custom libraries and adducts for AccurateMassSearch, the min/max fragment mass-to-charge to be able to restrict the mass of the transitions and the path to the PyProphet[6,7] executable. The DDA data is used for feature detection, adduct grouping, accurate mass search and forwarded to the AssayGeneratorMetabo. The constructed target library is used by the decoy generation node, which will call various python scripts to parse and reformat the input. In addition, Passatutto[3] is called and the target-decoy assay library exported. The assay library is used in combination with the SWATH-MS for OpenSWATH[37] and will be automatically analysed. PyProphet uses the results for scoring and exports a list of peak groups with associated FDR and quantitative values. For detailed parameters please check the workflow (.knwf).

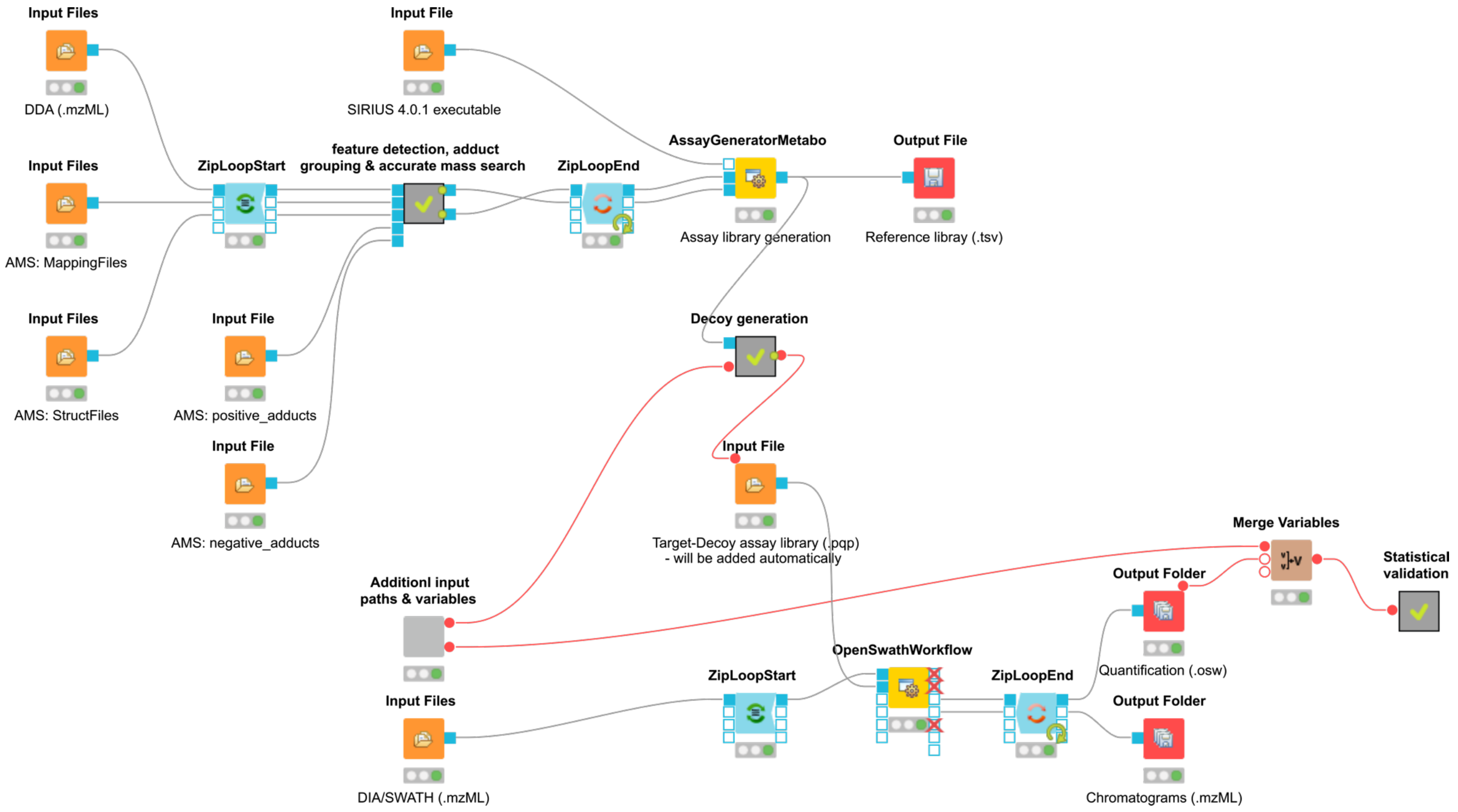

Supplementary Figure. 21 | Example workflow for the usage of the DIAMetAlyzer Pipeline in KNIME. Inputs are the SWATH-MS data in profile mode (.mzML), a path for saving the new target-decoy assay library, the SIRIUS 4.0.1 executable, the DDA data (.mzML), custom libraries and adducts for AccurateMassSearch, the min/max fragment mass-to-charge to be able to restrict the mass of the transitions and the path to the PyProphet executable. The DDA data is used for feature detection, adduct grouping, accurate mass search and forwarded to the AssayGeneratorMetabo. The constructed target library is used by the decoy generation node, which calls various python scripts to parse, reformat the input, call Passatutto and export the target-decoy assay library. The target-decoy assay library is processed with the SWATH-MS data in OpenSWATH. The results are used by PyProphet for scoring and output a list of metabolites with their respective q-value and quantitative information.

Evaluation of the identification performance

The top 20 features with the highest significant differences between groups based on DIAMetAlyzer + Unknown quantification were used to assess the identification performance (Supplementary Table 4). Within DIAMetAlyzer the molecular formula for features without prior MS1 identification was determined via SIRIUS. In most of these cases, no MS2 spectral library match was detected in the GNPS database using the MASST Search (workflow release version 27), but in almost all cases a mirror match was detected stemming from another *homo sapiens* dataset, which suggests a valid feature without spectral library entries. Following such cases could potentially lead to the identification of new compounds which are related to the biological question. Further three MS1 identifications were directly validated by GNPS spectral library search. Other MS1 identifications could not be validated by GNPS, either since no matching MS2 spectrum could be found or MS2 spectral library search found other possible identification based on the spectrum with a cosine similarity between 0.72 - 0.92. As in most metabolomics studies, the identification of compounds and their biological influence must be re-evaluated in follow up studies.

Supplementary Table 4 | Identification of the top 20 features based on DIAMetAlyzer + Unknown

Features	Identification (MS1 or de-novo)	GNPS (spectral library search)	Link to job
1292_C34H51N11O11_[M+H]+	UNKNOWN_595	No Hits in MS2 (GNPS), but community matches	https://gnps.ucsd.edu/ProteoSAFe/status.jsp?task=e81cc1216b5947cdb16dfb5a7f1a36e6
1376_C22H26O6_[M+H]+	Porson,IsogingerenoneB,GingerenoneB,?,?	C17H22O10 - cosine 0.84 - 1-O-Sinapoyl-beta-D-glucose	https://gnps.ucsd.edu/ProteoSAFe/status.jsp?task=2c21a2d7b9a64890864450b47bc403c9
1385_C22H29NO6_[M+H]+	UNKNOWN_619	No Hits in MS2 (GNPS), but community matches	https://gnps.ucsd.edu/ProteoSAFe/status.jsp?task=3731682576c34bcdb2dcdbad71bdf45a
1118_C23H37O7P_[M+H]+	1-(5Z,8Z,11Z,14Z,17Z-eicosapentaenoyl)-glycero-3-phosphate,LysoPA(20:5(5Z,8Z,11Z,14Z,17Z)/0:0),1-(5Z,8Z,11Z,14Z,17Z-eicosapentaenoyl)-glycero-3-phosphate	No Hits in MS2 (GNPS)	https://gnps.ucsd.edu/ProteoSAFe/status.jsp?task=0222cc9ff8db45aa8ff53a1c7af64a17
382_C21H28N6O4S_[M+H]+	N-Desmethylsildenafil(UK-103,320)	No Hits in MS2 (GNPS)	https://gnps.ucsd.edu/ProteoSAFe/status.jsp?task=7be87fce1597468591538a00ef4ab352
228_C16H19N3O5_[M+H]+	Glutamyltryptophan,Tryptophyl-Glutamate,gamma-Glutamyltryptophan	Spectral Match to Trp-Glu from NIST14 - Data from Pieter Dorrestein	https://gnps.ucsd.edu/ProteoSAFe/status.jsp?task=85ee7e43284a43a2ab7717cc52d7d3ff
874_C38H42O4_[M+K]+	UNKNOWN_564	C33H36N4O6 - bilirubin - 0.82 cosine similarity	https://gnps.ucsd.edu/ProteoSAFe/status.jsp?task=fd36000610ce4cec8301d4ee26449458

1482_C12H18O4S2_[M+H]+	Isoprothiolane	No Hits in MS2 (GNPS)	https://gnps.ucsd.edu/ProteoSAFe/status.jsp?task=3f281e9239cf410eaaff373fa1751deb
532_C18H23N3O6_[M+H]+	Imidaprilat	No Hits in MS2 (GNPS)	https://gnps.ucsd.edu/ProteoSAFe/status.jsp?task=7e04a1df3e3d440e959207897581326a
502_C14H22ClN3O2_[M+H]+	Metoclopramide	HIT: Massbank: Metoclopramide|4-Amino-5-chloro-N-[2-(diethylamino)ethyl]-2-methoxybenzamide	https://gnps.ucsd.edu/ProteoSAFe/status.jsp?task=11cc03314f344b4a9e5b51efdfdae050
1234_C30H42O12_[M+Na]+	3-O(beta-D-glucopyranosyl)-3beta,5beta,14beta,16beta-tetrahydroxy-19-oxo-bufa-20,22-dienolide	? 3-[(3-Cholamidopropyl)dimethylammonio]-1-propanesulfonate from NIST14 - consine 0.73	https://gnps.ucsd.edu/ProteoSAFe/status.jsp?task=a4d585bc4ff44b0c8f583836c8251f33
1594_C28H50NO8P_[M+H]+	2-(11R-hydroxy-5Z,8Z,12E,14Z-eicosatetraenoyl)-sn-glycero-3-phosphocholine,2-(15R-hydroxy-5Z,8Z,12E,14Z-eicosatetraenoyl)-sn-glycero-3-phosphocholine,2-(15S-hydroxy-5Z,8Z,12E,14Z-eicosatetraenoyl)-sn-glycero-3-phosphocholine	No Hits in MS2 (GNPS), but community matches	https://gnps.ucsd.edu/ProteoSAFe/status.jsp?task=193fa5748add45c981845cc037fc27a8
598_C14H16N2O4_[M+H]+	N-lactoyl-Tryptophan	No Hits in MS2 (GNPS), but community matches	https://gnps.ucsd.edu/ProteoSAFe/status.jsp?task=87b4f973666f46af82d060ccc4985018
1524_C17H37NO2_[M+H]+	heptadecasphinganine	C16H33NO3 - Lauryl diethanolamide - cosine 0.92	https://gnps.ucsd.edu/ProteoSAFe/status.jsp?task=66031f1291984e5ab2de57b8a04d4ff3
280_C14H18N2O5_[M+H]+	gamma-Glutamylphenylalanine,Aspartame,Hydroxyprolyl-Tyrosine,Phenylalanyl-Glutamate,Tyrosyl-Hydroxyproline,Glutamylphenylalanine,4'-tert-Butyl-2',6'-dimethyl-3',5'-dinitroacetophenone,L-gamma-Glutamyl-beta-phenyl-beta-L-alanine	No Hits in MS2 (GNPS), but community matches	https://gnps.ucsd.edu/ProteoSAFe/status.jsp?task=610b621bf01340e6bd328e148935fe75
1073_C17H36O7_[M+H]+	UNKNOWN_500	No Hits in MS2 (GNPS)	https://gnps.ucsd.edu/ProteoSAFe/status.jsp?task=2ab6dcc7f0184f7d97a13d71051f63c9
472_C26H29N3O6_[M+H]+	Nicardipine	No Hits in MS2 (GNPS), but community matches	https://gnps.ucsd.edu/ProteoSAFe/status.jsp?task=daa5c6acdf6d490385241281af97b9b4
995_C20H39N5O4_[M+H]+	UNKNOWN_476	No Hits in MS2 (GNPS), but community matches	https://gnps.ucsd.edu/ProteoSAFe/status.jsp?task=8b00c287606c45ebb773e8b6ed545820
1552_C26H50NO8P_[M+H]+	1-(9Z-hexadecenoyl)-2-acetyl-sn-glycero-3-phosphocholine	C27H55N1O7P1 cosine 0.72	https://gnps.ucsd.edu/ProteoSAFe/status.jsp?task=8f1faf40c6a9400d8d536687ff896d51
1970_C26H54NO6P_[M+H]+	LysoPC(P-18:0),1-(11Z-octadecenyl)-sn-glycero-3-phosphocholine,1-(9Z-octadecenyl)-sn-glycero-3-phosphocholine,1-(1Z-octadecenyl)-sn-glycero-3-phosphocholine	Spectral Match to 1-(1Z-Octadecenyl)-sn-glycero-3-phosphocholine from NIST14 - consine 0.92	https://gnps.ucsd.edu/ProteoSAFe/status.jsp?task=7dfc1cadabc742508458c64614953217

Supplementary References

1. Benjamini, Y. & Hochberg, Y. Controlling the False Discovery Rate: A Practical and Powerful Approach to Multiple Testing. *Journal of the Royal Statistical Society: Series B (Methodological)* vol. 57 289–300 (1995).
2. Elias, J. E. & Gygi, S. P. Target-decoy search strategy for increased confidence in large-scale protein identifications by mass spectrometry. *Nat. Methods* 4, 207–214 (2007).
3. Scheubert, K. *et al.* Significance estimation for large scale metabolomics annotations by spectral matching. *Nat. Commun.* 8, 1494 (2017).
4. Wang, X. *et al.* Target-Decoy-Based False Discovery Rate Estimation for Large-Scale Metabolite Identification. *J. Proteome Res.* 17, 2328–2334 (2018).
5. Reiter, L. *et al.* mProphet: automated data processing and statistical validation for large-scale SRM experiments. *Nat. Methods* 8, 430–435 (2011).
6. Teleman, J. *et al.* DIANA—algorithmic improvements for analysis of data-independent acquisition MS data. *Bioinformatics* 31, 555–562 (2015).
7. Rosenberger, G. *et al.* Statistical control of peptide and protein error rates in large-scale targeted data-independent acquisition analyses. *Nat. Methods* 14, 921–927 (2017).
8. Storey, J. D. & Tibshirani, R. Statistical significance for genomewide studies. *Proc. Natl. Acad. Sci. U. S. A.* 100, 9440–9445 (2003).
9. Bruderer, T. *et al.* Metabolomic spectral libraries for data-independent SWATH liquid chromatography mass spectrometry acquisition. *Anal. Bioanal. Chem.* 410, 1873–1884 (2018).
10. Röst, H., Malmström, L. & Aebersold, R. A computational tool to detect and avoid redundancy in selected reaction monitoring. *Mol. Cell. Proteomics* 11, 540–549 (2012).
11. Sherman, J., McKay, M. J., Ashman, K. & Molloy, M. P. How specific is my SRM?: The issue of precursor and product ion redundancy. *Proteomics* 9, 1120–1123 (2009).
12. Sherman, J., McKay, M. J., Ashman, K. & Molloy, M. P. Unique ion signature mass spectrometry, a deterministic method to assign peptide identity. *Mol. Cell. Proteomics* 8, 2051–2062 (2009).
13. Shanthamoorthy, P., Young, A. & Röst, H. Analyzing Assay Specificity in Metabolomics Using Unique Ion Signature Simulations. *Anal. Chem.* 93, 11415–11423 (2021).
14. Schubert, O. T. *et al.* Building high-quality assay libraries for targeted analysis of SWATH MS data. *Nat. Protoc.* 10, 426–441 (2015).
15. Tsou, C.-C. *et al.* DIA-Umpire: comprehensive computational framework for data-independent acquisition proteomics. *Nat. Methods* 12, 258–64, 7 p following 264 (2015).
16. Benton, H. P., Want, E. J. & Ebbels, T. M. D. Correction of mass calibration gaps in liquid chromatography-mass spectrometry metabolomics data. *Bioinformatics* 26, 2488–2489 (2010).

17. Kuhl, C., Tautenhahn, R., Böttcher, C., Larson, T. R. & Neumann, S. CAMERA: an integrated strategy for compound spectra extraction and annotation of liquid chromatography/mass spectrometry data sets. *Anal. Chem.* 84, 283–289 (2012).
18. Gupta, S., Ahadi, S., Zhou, W. & Röst, H. DIAlignR Provides Precise Retention Time Alignment Across Distant Runs in DIA and Targeted Proteomics. *Mol. Cell. Proteomics* 18, 806–817 (2019).
19. Alka, O. & Shanthamoorthy, P. DIAMetAlyzer for automated false discovery rate controlled analysis for data independent acquisition in metabolomics. *oliveralka/DIAMetAlyzer_additional_code* https://doi.org/10.5281/zenodo.5913236 (2022) doi:10.5281/zenodo.5913236.
20. Kenar, E. *et al.* Automated Label-free Quantification of Metabolites from Liquid Chromatography–Mass Spectrometry Data. *Mol. Cell. Proteomics* 13, 348–359 (2014).
21. Pluskal, T., Castillo, S., Villar-Briones, A. & Oresic, M. MZmine 2: modular framework for processing, visualizing, and analyzing mass spectrometry-based molecular profile data. *BMC Bioinformatics* 11, 395 (2010).
22. Melamud, E., Vastag, L. & Rabinowitz, J. D. Metabolomic analysis and visualization engine for LC-MS data. *Anal. Chem.* 82, 9818–9826 (2010).
23. Pang, Z. *et al.* MetaboAnalyst 5.0: narrowing the gap between raw spectra and functional insights. *Nucleic Acids Res.* 49, W388–W396 (2021).
24. Mitchell, S. L. *et al.* The Carnitine Shuttle Pathway is Altered in Patients With Neovascular Age-Related Macular Degeneration. *Invest. Ophthalmol. Vis. Sci.* 59, 4978–4985 (2018).
25. Hou, X.-W., Wang, Y. & Pan, C.-W. Metabolomics in Age-Related Macular Degeneration: A Systematic Review. *Invest. Ophthalmol. Vis. Sci.* 61, 13 (2020).
26. Zhu, W. *et al.* Identification of lncRNAs involved in biological regulation in early age-related macular degeneration. *Int. J. Nanomedicine* 12, 7589–7602 (2017).
27. Reichenbach, A. & Bringmann, A. Purinergic signaling in retinal degeneration and regeneration. *Neuropharmacology* 104, 194–211 (2016).
28. Cho, B.-J., Heo, J. W., Kim, T. W., Ahn, J. & Chung, H. Prevalence and risk factors of age-related macular degeneration in Korea: the Korea National Health and Nutrition Examination Survey 2010-2011. *Invest. Ophthalmol. Vis. Sci.* 55, 1101–1108 (2014).
29. Yildirim, Z., Ucgun, N. I. & Yildirim, F. The role of oxidative stress and antioxidants in the pathogenesis of age-related macular degeneration. *Clinics* 66, 743–746 (2011).
30. Merle, B. M. J. *et al.* Circulating omega-3 Fatty acids and neovascular age-related macular degeneration. *Invest. Ophthalmol. Vis. Sci.* 55, 2010–2019 (2014).
31. Bazan, N. G. Neuroprotectin D1-mediated anti-inflammatory and survival signaling in stroke, retinal degenerations, and Alzheimer's disease. *J. Lipid Res.* 50 Suppl, S400-5 (2009).
32. SanGiovanni, J. P. & Chew, E. Y. The role of omega-3 long-chain polyunsaturated fatty acids in health and disease of the retina. *Prog. Retin. Eye Res.* 24, 87–138 (2005).

33. Schymanski, E. L. *et al.* Identifying small molecules via high resolution mass spectrometry: communicating confidence. *Environ. Sci. Technol.* 48, 2097–2098 (2014).

34. Berthold, M. R. *et al.* KNIME: The Konstanz Information Miner. *Data Analysis, Machine Learning and Applications* 319–326 (2008) doi:10.1007/978-3-540-78246-9_38.

35. Fillbrunn, A. *et al.* KNIME for reproducible cross-domain analysis of life science data. *J. Biotechnol.* 261, 149–156 (2017).

36. Dührkop, K. *et al.* SIRIUS 4: a rapid tool for turning tandem mass spectra into metabolite structure information. *Nat. Methods* 16, 299–302 (2019).

37. Röst, H. L. *et al.* OpenSWATH enables automated, targeted analysis of data-independent acquisition MS data. *Nat. Biotechnol.* 32, 219–223 (2014).

Supplementary Note

Software Requirements

System requirements
DIAMetAlyzer as KNIME workflow runs on all systems (Linux, Windows, macOS)

Tested on:
MacOS (10.15)
Windows 10 (19042.630)
Linux (20.4)

Installation instructions (~ 5-15 min)
A short overview and instructions on how to install KNIME can be found here: https://www.openms.de/comp/diametalyzer/

Demo (~ 3-5h)
Please follow the steps indicated in Chapter 8 - OpenSWATH for Metabolomics in the handout to run the workflow using KNIME:
https://abibuilder.informatik.uni-tuebingen.de/archive/openms/Tutorials/Handout/release/2.6.0/handout.pdf

Example data can be acquired here:
https://abibuilder.informatik.uni-tuebingen.de/archive/openms/Tutorials/Data/DIAMetAlyzer/

An updated workflow and an example output file (.tsv) can be found here:
https://abibuilder.informatik.uni-tuebingen.de/archive/openms/Tutorials/Data/DIAMetAlyzer_addition/

General
The code is available in the OpenMS Framework
https://github.com/OpenMS/OpenMS

In addition, third party tools are used in the workflow such as:
KNIME 4.2.1 (https://www.knime.com/)
SIRIUS 4.0.1 (https://bio.informatik.uni-jena.de/software/sirius-4-0-1/)
Passatutto (https://github.com/boecker-lab/passatuto)
PyProphet (https://github.com/PyProphet/pyprophet)

To improve reproducibility of the study, all parameters are stored in the KNIME workflow. In addition, further scripts and parameters are available here:
https://github.com/oliveralka/DIAMetAlyzer_additional_code

Supplementary Table. 5 | Example output of the workflow (.tsv).

Transition Group id	decoy	run_id	filename	RT	assay_rt	delta_rt	assay_RT	delta_RT	id	sum_formula	compound_name
0	0	4.79E+18	PestMix1_8Step10Plasma1SWATH20-50.mzML	161.353	146.16	15.193	146.16	15.193	3.9619E+18	C4H10NO3PS	Acephate
0	0	6.9146E+18	PestMix1_8Step10Plasma2SWATH20-50.mzML	142.949	146.15958	-3.21058	146.16	-3.211	7.9878E+18	C4H10NO3PS	Acephate
0	0	8.7002E+18	PestMix1_8Step10Plasma3SWATH20-50.mzML	139.957	146.15994	-6.20294	146.16	-6.203	8.7785E+18	C4H10NO3PS	Acephate
0	0	3.9989E+18	PestMix1_8Step1Plasma1SWATH20-50.mzML	143.845	146.16046	-2.31546	146.16	-2.315	3.9869E+18	C4H10NO3PS	Acephate
0	0	7.51E+18	PestMix1_8Step1Plasma2SWATH20-50.mzML	145.487	146.159935	-0.672935	146.16	-0.673	7.7789E+18	C4H10NO3PS	Acephate

Adducts	Charge	mz	Intensity	aggr_prec_Peak_Area	aggr_prec_Peak_Apex	leftWidth	rightWidth	peak_group_rank	d_score	m_score
M+H+	1	184.019177	10.699	51.026001	62	159.707001	162.998993	1	-0.3057201	0.16474964
M+H+	1	184.019177	32.097	0	0	140.166	144.279999	1	0.89540231	0.05848996
M+H+	1	184.019177	22.75	107.813004	131	138.311005	141.602997	1	0.28296408	0.10950692
M+H+	1	184.019177	41942.8	65994.6621	20587	140.779999	149.832001	1	2.4779129	0.00761936
