# Supplementary material for: DIAMetAlyzer allows automated false-discovery rate-controlled analysis for data-independent acquisition in metabolomics
Source: Nat Commun. 2022 Mar 15;13:1347. doi: 10.1038/s41467-022-29006-z (PMC8924252; doi:10.1038/s41467-022-29006-z)
Supplement: Supplementary file 2 — Reporting Summary [file 41467_2022_29006_MOESM2_ESM.pdf]

nature portfolio

Corresponding author(s): Oliver Alka, Hannes Röst, Oliver Kohlbacher

Last updated by author(s): Feb 1, 2022

Reporting Summary

Nature Portfolio wishes to improve the reproducibility of the work that we publish. This form provides structure for consistency and transparency in reporting. For further information on Nature Portfolio policies, see our Editorial Policies and the Editorial Policy Checklist.

Statistics

For all statistical analyses, confirm that the following items are present in the figure legend, table legend, main text, or Methods section.

n/a	Confirmed	
☐	☒	The exact sample size (n) for each experimental group/condition, given as a discrete number and unit of measurement
☐	☒	A statement on whether measurements were taken from distinct samples or whether the same sample was measured repeatedly
☐	☒	The statistical test(s) used AND whether they are one- or two-sided<br>*Only common tests should be described solely by name; describe more complex techniques in the Methods section.*
☒	☐	A description of all covariates tested
☐	☒	A description of any assumptions or corrections, such as tests of normality and adjustment for multiple comparisons
☐	☒	A full description of the statistical parameters including central tendency (e.g. means) or other basic estimates (e.g. regression coefficient) AND variation (e.g. standard deviation) or associated estimates of uncertainty (e.g. confidence intervals)
☐	☒	For null hypothesis testing, the test statistic (e.g. F, t, r) with confidence intervals, effect sizes, degrees of freedom and P value noted<br>*Give P values as exact values whenever suitable.*
☒	☐	For Bayesian analysis, information on the choice of priors and Markov chain Monte Carlo settings
☒	☐	For hierarchical and complex designs, identification of the appropriate level for tests and full reporting of outcomes
☒	☐	Estimates of effect sizes (e.g. Cohen's d, Pearson's r), indicating how they were calculated

Our web collection on statistics for biologists contains articles on many of the points above.

Software and code

Policy information about availability of computer code

Data collection	SWATH Variable Window Calculator (SCIEX) 1.1; Proteowizard (msconvert, qtofpeakpicker) 3.0.19347;
Data analysis	KNIME 4.1.3; OpenMS 2.5.0; OpenMS (14f627e); pyOpenMS 2.5.0; SIRIUS 4.0.1; SIRIUS 4.5.0; passatutto 1.0; Pyprophet 2.1.5; Skyline 19.1.0.193; DIAlignR (4e94203); MetaboDIA 1.3; DIAUmpire 2.1.6; MS-DIAL 4.60; MetaboAnalyst (5.0); MASST Search (workflow release 27); ABF Converter (1.0); Limma (3.44.3); Additional custom code, which is partially integrated in the workflow (python 3.7, R 3.6 - https://github.com/oliveralka/DIAMetAlyzer_additional_code). KNIME Workflow and OpenMS Tutorial: https://www.openms.de/comp/diametalyzer/ and https://github.com/OpenMS/Tutorials.

For manuscripts utilizing custom algorithms or software that are central to the research but not yet described in published literature, software must be made available to editors and reviewers. We strongly encourage code deposition in a community repository (e.g. GitHub). See the Nature Portfolio guidelines for submitting code & software for further information.

Data

Policy information about availability of data

All manuscripts must include a data availability statement. This statement should provide the following information, where applicable:

- Accession codes, unique identifiers, or web links for publicly available datasets
- A description of any restrictions on data availability
- For clinical datasets or third party data, please ensure that the statement adheres to our policy

The dataset is publicly available in MetaboLights under accession MTBLS1108 (https://www.ebi.ac.uk/metabolights/MTBLS1108). Comparison with MetaboDIA was performed using publicly available data MTBLS417 (https://www.ebi.ac.uk/metabolights/MTBLS417). Databases used were HMDB 4.0 (https://hmdb.ca/), LIPIDMAPS (092020) (https://www.lipidmaps.org/)

Field-specific reporting

Please select the one below that is the best fit for your research. If you are not sure, read the appropriate sections before making your selection.

[x] Life sciences [ ] Behavioural & social sciences [ ] Ecological, evolutionary & environmental sciences

For a reference copy of the document with all sections, see nature.com/documents/nr-reporting-summary-flat.pdf

Life sciences study design

All studies must disclose on these points even when the disclosure is negative.

Sample size	n=30, 30 DIA samples with APM spiked-in human blood plasma, 10-step dilution series. We chose a dilution series with 10 steps of four-fold dilution as this covers the expected dynamic range of a mass spectrometer. Three repetitions are a common standard in the field.
Data exclusions	No data was excluded from the analysis
Replication	Three replicates per dilution step were created. All attempts at replication were successful based on the coefficient of variation below 0.2 and a manual validation in Skyline (19.1.0.193).
Randomization	No randomization was applied due to the use of a dilution series (Measured from lowest to highest concentration). Since this is a spike-in ground truth dataset consists of one sample group (at different concentrations) measured continuously in a very short time frame we did not expect any covariates.
Blinding	Blinding was not relevant for this benchmarking study, since we compared different softwares to the same sample group (spike-in) data.

Reporting for specific materials, systems and methods

We require information from authors about some types of materials, experimental systems and methods used in many studies. Here, indicate whether each material, system or method listed is relevant to your study. If you are not sure if a list item applies to your research, read the appropriate section before selecting a response.

Materials & experimental systems

n/a	Involved in the study	
[x]	[ ]	Antibodies
[x]	[ ]	Eukaryotic cell lines
[x]	[ ]	Palaeontology and archaeology
[x]	[ ]	Animals and other organisms
[x]	[ ]	Human research participants
[x]	[ ]	Clinical data
[x]	[ ]	Dual use research of concern

Methods

n/a	Involved in the study	
[x]	[ ]	ChIP-seq
[x]	[ ]	Flow cytometry
[x]	[ ]	MRI-based neuroimaging
